# Mechanism of Anti-Inflammatory and Antibacterial Effects of QingXiaoWuWei Decoction Based on Network Pharmacology, Molecular Docking and *In Vitro* Experiments

**DOI:** 10.3389/fphar.2021.678685

**Published:** 2021-07-15

**Authors:** Qian Zhang, Xue Li, Jun Li, Yuxia Hu, Jing Liu, Fang Wang, Wei Zhang, Fuhou Chang

**Affiliations:** ^1^ The Center for New Drug Safety Evaluation and Research, Inner Mongolia Medical University, Hohhot, China; ^2^ The Center for New Drug Screening Engineering and Research of Inner Mongolia Autonomous Region, Inner Mongolia Medical University, Hohhot, China; ^3^ College of Pharmacy, Inner Mongolia Medical University, Hohhot, China

**Keywords:** QingXiaoWuWei Decoction, anti-inflammatory, antibacterial, network pharmacology, molecular docking, HPLC-Q-Exactive-MS

## Abstract

**Background and Aim:** QingXiaoWuWei Decoction (QXWWD) is a traditional Chinese medicine that is commonly used in clinical settings to treat inflammatory and bacterial diseases. However, there is still a lot to learn about its molecular mechanism. A network pharmacology approach was applied to investigate the pharmacological mechanisms of QXWWD in inflammation treatment.

**Methods:** The basic mechanisms involved in the anti-inflammatory and antibacterial potentials of QXWWD were identified using network pharmacology and molecular docking. The principal components of QXWWD were identified by the HPLC-Q-Exactive-MS method. The antibacterial bioactivity of QXWWD was further investigated using the Kirby-Bauer disc diffusion method and the determination of the minimum inhibitory concentration. The anti-inflammatory activity of QXWWD was evaluated using mice ear swelling test, RAW264.7 cell culture, and pro-inflammatory cytokines measurement. Skin irritation and HE staining were employed to evaluate the safety of QXWWD topical use and to depict the drug’s potential therapeutic function. The hub genes and signaling pathways associated with inflammatory and bacterial diseases were validated by western blot in addition to biochemical and pathological markers.

**Results:** Our findings revealed that the ethanolic extract of QXWWD had a strong inhibitory effect against *Staphylococcus aureus*, *Enterococcus faecalis*, *and Streptococcus pneumoniae.* Meanwhile, QXWWD was potentially effective in suppressing ear swelling, elevated white blood cell counts, and the TNF-α, IL-1, and IL-6 levels. According to skin irritation, QXWWD was found to be safe when tested for topical application. The results of HE staining showed that the possible therapeutic role of QXWWD was related to the change in skin microstructure. Also, the network pharmacology, molecular docking as well as Q-Exactive-MS and HPLC analysis suggested that the synergistic effect of quercetin, luteolin and other ingredients could serve as main contributor of QXWWD for its anti-inflammatory and antibacterial activities. Moreover, the JUN, MAPK1, RELA, NFKBIA, MYC, and AKT1 were the potential identified key targets, and MAPK/PI3K/Akt was among the possibly involved signaling pathways in the anti-inflammatory and antibacterial activities of QXWWD.

**Conclusions:** From a therapeutic standpoint, QXWWD may be a promising antibacterial and anti-inflammatory agent for the treatment of bacterial, acute, and chronic dermatitis.

## Introduction

Inflammation is one of the most common and complex reactions, which mostly occurs due to the cell damage or death caused by certain chemicals or bacterial infection, resulting in the release of inflammatory cytokines like IL-1, IL-6, and TNF-α from the macrophages and leukocytes (Zhao Y et al., 2014; Wang et al., 2015; Paolucci et al., 2018; Ahmad et al., 2020; Diaz et al., 2021). For the management of such inflammatory events, NSAIDs (Non-steroidal anti-inflammatory drugs) are the widely used therapeutics, and they play a prominent role among anti-inflammatory drugs due to their strong anti-inflammatory potentials (Bacchi et al., 2012; Dona et al., 2016; Marquez-Lara et al., 2016; Bindu et al., 2020; Zappavigna et al., 2020). Among them, ibuprofen and aspirin have emerged as effective anti-inflammatory drugs that could potentially inhibit prostaglandin biosynthesis by inhibiting the function of the cyclooxygenase (COX) enzyme (Harris et al., 2005; Renda et al., 2006; Harris et al., 2012). Long-term use of NSAIDs, on the other hand, is linked to a variety of side effects, including bleeding, coagulopathy, interstitial nephritis, gastrointestinal mucosa damage, and allergic reactions due to very low prostaglandin levels (Harirforoosh et al., 2013; Marjoribanks et al., 2015). Traditional Chinese herbal medicines have a broad antibacterial range and low drug resistance, which makes them ideal for treating inflammation (Zhao CQ et al., 2014; Cheng and Merz, 2016). In one study carried by (Zhang L et al., 2013), 58 kinds of traditional Chinese herbal medicines were screened out based on their traditional usage and the existing literature. The antimicrobial effects of their ethanol extracts were assessed against yeast (*Candida albicans*), mold (*Aspergillus fumigatus*), Gram-positive bacteria (*Staphylococcus aureus*) as well as Gram-negative bacteria (*Acinetobacter baumanniiand* and *Pseudomonas aeruginosa*). The results indicated that 15 extracts showed an anti-fungal effect while 23 extracts exhibited an antibacterial effect. Further, eight extracts exhibited both anti-fungal and antibacterial effects. For example, *Polygonum cuspidatum, Eucommia ulmoides, Uncaria rhyncophylla,* and *Poria cocos* showed effects against both fungal and bacterial strains, indicating their broad spectrum of activities. On the other hand, due to the multi-component characteristics, many small molecules with good binding affinity were identified from the Traditional Chinese Medicine database as potential candidates. Herbal therapies are thus useful in combating fungal and bacterial resistance because they have low drug resistance, high effectiveness, and are widely available. For the reasons mentioned above, they have attracted the interest of researchers looking into the use of herbal medicines to treat a variety of diseases. When combined with the broad antibacterial spectrum, traditional Chinese herbal medicines are expected to be formulated as therapeutic drugs for the treatment of inflammatory and bacterial diseases.

QingXiaoWuWei Decoction (QXWWD) is traditional Chinese medicine and is composed of *Sophorae flavescentis radix* (dried radix of *Sophora flavescens* Aiton), *Fructus cnidii* (dried and ripe fructus of *Cnidium monnieri* (L.) Cusson), *Rhei Radix et Rhizoma* (dried rhizomia of *Rheum palmatum* L), *Artemisia Argyi Folium* (dried folium of *Artemisia argyi* H. Lév. and Vaniot), and *Borneolum* (extraction of branch and folium of *Cinnamomum camphora* (L.) J. Presl). To prepare a typical traditional decoction, a mixture of *Sophorae Flavescentis Radix*, *Fructus Cnidii*, *Rhei Radix et Rhizoma*, and *Artemisia Argyi Folium* (3, 3, 3, 3 g) is soaked in water (usually with adding less strong liquor) for ∼0.5–1 h before a first boiling step follows. After filtration, the herbal material is reextracted twice with less water and the combined extracts are collected. The full extract is then added with 0.15 g *Borneolum* to yield the final QXWWD preparation for one-day use.

In Traditional Chinese Medicine, *Sophorae Flavescentis Radix*, *Fructus Cnidii*, *Rhei Radix et Rhizoma*, *Artemisia Argyi Folium*, and *Borneolum* can be used alone or in combination for the treatment of inflammatory diseases. For instance, the dried radix of *Sophorae Flavescentis Radix* has anti-inflammatory, antibacterial, antiviral, anticancer, and antifibrotic properties, and can also be used to treat various immunological diseases (Ma et al., 2018). The predominant compounds in *Fructus Cnidii* and *Rhei Radix et Rhizoma* have antibacterial, anti-inflammatory, antifibrotic, anticancer, and antioxidant activities (Zhong et al., 2017; Espinosa et al., 2020). *Artemisia Argyi Folium* is traditionally used in South East Asia for the treatment of various inflammatory diseases, such as dermatitis, arthritis, and bronchitis (Chen et al., 2017). *Borneolum*, a widely used food, and cosmetics additive, has antibacterial, analgesic, anti-inflammatory and penetration promoting effects (Dai et al., 2009). In summary, the clinical application and existing literature demonstrate that QXWWD therapy is both internally and externally safe and effective.

**FIGURE 1:**
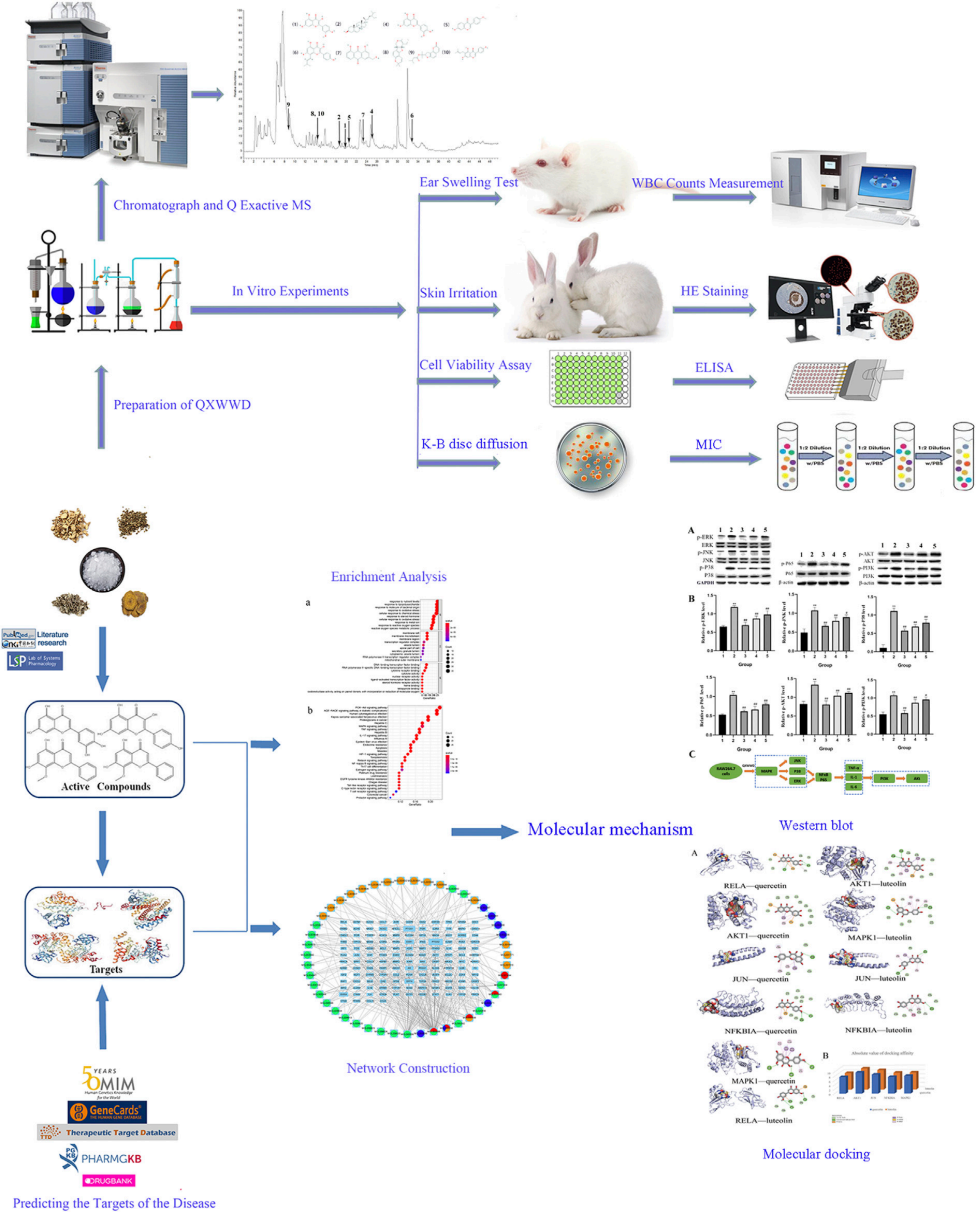
Workflow for QXWWD treatment of inflammatory and bacterial diseases.

In China, QXWWD has long been used to treat inflammatory conditions such as acute and chronic dermatitis. However, there is still a lot to learn about its molecular mechanism. The whole review and systematic characteristics of network pharmacology are of great significance for assessing traditional Chinese medicines, the theory related to syndrome differentiation and synergy in the Chinese medicines developed among various components, channels and targets as well. This, in turn, provides new insights into the fundamental studies of complex traditional Chinese medicine systems. Therefore, the compounds in QXWWD, their anti-inflammatory potentials, and the underlying molecular mechanism of such effects could easily be evaluated by adopting network pharmacology and molecular docking. Considering the therapeutic effects of QXWWD, the anti-inflammatory and antibacterial effects and related mechanisms were verified by *in vitro* experiments using bacteria, cells, and animal models. The workflow of the study is shown in Figure 1.

## Materials and Methods

### Chemicals and Materials

*Sophorae Flavescentis Radix* (“Kushen” in Chinese, KS, batch number 190601), dried radix of *Sophora flavescens* Aiton, *Fructus Cnidii* (“Shechuangzi” in Chinese, SCZ, batch number 191001), dried and ripe fructus of *Cnidium monnieri* (L.) Cusson, *Rhei Radix et Rhizoma* (“Dahuang” in Chinese, DH, batch number 190901), dried rhizomia of *Rheum palmatum* L, *Artemisia Argyi Folium* (“Aiye” in Chinese, AY, batch number 190501), dried folium of *Artemisia argyi* H. Lév. and Vaniot, *Borneolum* (“Bingpian” in Chinese, BP, batch number 191001), extraction of branch and folium of *Cinnamomum camphora* (L.) J. Presl were all procured from the Anguo medicine market (Baoding, China). The whole plant materials of the five herbal materials were obtained from their original source and the botanic identification was carried out by Professor Bi Qu (Inner Mongolia Medical University, Hohhot, China). The specimens were deposited at the herbarium of medicinal plants at College of Pharmacy, Inner Mongolia Medical University, until further processing.

The KM mice (male and female half, 5–6 weeks old, weight range: 18–22 g) were procured from Beijing Weitonglihua Laboratory Animal Co, Ltd. (production license number: SCXK (Jing) 2012–0,001, Beijing, China). Twelve rabbits (male and female half, 2 months old, weight range: 1.8–2.2 kg) were obtained from Suzhou Huqiao Biotechnology Co, Ltd. (production license number: SCXK (Su) 2015–0,002, Suzhou, China). Three strains of *Staphylococcus aureus* with different drug resistance (ATCC29213, ATCC25923, ATCC43300), *Enterococcus faecalis* (ATCC29212), *Streptococcus pneumoniae* (ATCC49619), *Pseudomonas aeruginosa* (ATCC27853), *Streptococcus pyogenes* (ATCC19615), *Klebsiella pneumoniae* (ATCC700603), *Escherichia coli* (ATCC25922), *Enterobacter cloacae* (ATCC700323) and RAW264.7 cells were procured from ATCC (Rockville, MD, United States). The DMEM and Fetal bovine serum (FBS) were acquired from GIBCO (Grand Island, NY, United States). MH agar plates were obtained from Qingdao Haibo Biotechnology Co, Ltd. (Qingdao, China). ELISA kits and lipopolysaccharide (LPS) were procured from Soleibao Technology Co, Ltd. (Beijing, China). CCK8 kits were procured from Meilun Biotechnology Co, Ltd. (Dalian, China). Aspirin was purchased from Langtze Biomedical Technology (Nanjing, China). The thin layer silica gel plates were procured from Qingdao Ocean Chemical Co, Ltd. (Qingdao, China). All of the solvents used in the experiments were of an analytical quality (Tianjin Fuyu Fine Chemical Co, Ltd, Tianjin, China). Methanol and formic acid (HPLC-grade) were procured from Fisher Chemicals (Fisher, United States). All the animal studies comply with the 3 R (Heinrich M et al., 2020) of animal research and were approved by the Animal Ethics Committee of Inner Mongolia Medical University (YKD202002060).

### Identification and Screening of Main Chemical Compounds and Protein Targets of QXWWD

The database and analysis Platform (TCMSP, https://tcmspw.com/tcmsp.php) of Traditional Chinese Medicine Systems Pharmacology was used for the authentication of main chemical compounds and the protein targets linked with these compounds of the five herbs in QXWWD. The main chemical compounds of QXWWD were opted according to the established criterion of both OB ≥ 30% and DL ≥ 0.18. To further evaluate the network pharmacology, the protein targets such as gene ID and gene names were standardized by employing UniProtKB (http://www.uniprot.org).

### Predicting the Targets of the Disease

The keywords “inflammation and bacteria” were utilized for screening the inflammation and bacteria-associated targets while searching the GeneCards database (https://www.genecards.org/), DrugBank database (https://go.drugbank.com/), TTD database (http://db.idrblab.net/ttd//), PharmGkb database (https://www.pharmgkb.org/), and OMIM database (https://omim.org/). The most frequent targets of QXWWD and disease were then assembled using the R platform.

### Construction and Analysis of the Interaction Network

The common targets of QXWWD and disease were introduced into the STRING database (https://string-db.org/cgi/input.pl) to construct the PPI (protein-protein interaction) network. The species was selected as “*Homo sapiens*,” the lowest interaction score was greater than “0.900” and the PPI network was exported after hiding the free points. Cytoscape3.8.0 was employed to build and image the PPI network. CytoNCA, a network plug-in in Cytoscape, was utilized for screening the hub genes of the network by experimental data available for protein interaction networks, such as Closeness (CC), Betweenness (BC), Eigenvector (EC), Degree (DC), and Local Average Connectivity-based method (LAC). The level of these five parameters represented the topological significance of a node in a network, i.e., the higher the value, the more significant the node is. The targets with a degree greater than the median were chosen as the hub genes and were considered for further molecular docking.

### GO and KEGG Pathway Enrichment Analysis

To deeply explore the pathways and biological process of the network, GO (Gene Ontology) and KEGG (Kyoto Encyclopedia of Genes and Genomes) pathway analyses were conducted by employing the cluster profiler package in the R platform. GO enrichment analysis encompassed cellular components (CC), biological processes (BP), and molecular functions (MF). The statistical significance threshold of enrichment analysis was established at *p* ≤ 0.05.

### Molecular Docking

PubChem database (https://pubchem.ncbi.nlm.nih.gov/) was used for acquiring the 2D structures of the basic compounds. Then, Chem3D was used to process and transfer the 2D structure of the main compounds into a 3D structure, which was saved as ligands in PDBQT format. The 3D structure of the desired molecules was retrieved from the PDB database (https://www.rcsb.org/). PyMol was utilized for eliminating the water molecules, added the nonpolar hydrogen for the structure, and saved it as a PDBQT file. Autodock Vina 1.1.2 was employed to dock ligands with target molecules, respectively. The files after molecular docking were visualized by DiscoveryStudio2020 software.

### Preparation of QXWWD

Equivalent weight (18 g) of *Sophorae Flavescentis Radix*, *Fructus Cnidii*, *Rhei Radix et Rhizoma*, and *Artemisia Argyi* Folium were soaked in 75% ethanol (w/v = 1: 10) for 30 min, then refluxed twice (1 h each time), and finally filtered. The resultant filtrate was collected and concentrated to prepare the final concentration equivalent to 1 g raw herb/mL. *Borneolum* (0.9 g) was added to the filtrate for the Q Exactive MS analysis and the antibacterial activity test. The filtrate was concentrated in a rotary evaporator at 40°C, freeze-dried, and preserved for xylene-induced ear swelling test, cell culture viability assay, inflammatory factors measurement, western blot assay and skin irritation test.

### Thin Layer Chromatography

Following the method of Chinese Pharmacopoeia 2015, the identification of *Sophorae Flavescentis Radix*, *Fructus Cnidii*, *Rhei Radix et Rhizoma*, *Artemisia Argyi Folium*, and *Borneolum* was conducted by employing the thin layer chromatography method.

### Identification of the Main Chemical Components in QXWWD by Chromatograph and Q Exactive MS.

The extract of QXWWD was centrifuged at 1 × 10^4^ rpm for 15 min, and the supernatant was diluted with 50% methanol to appropriate concentrations. The resultant solution was filtered through a 0.22 μm filter for the HPLC-Q-Exactive-MS analysis.

HPLC conditions: Analyses were conducted on the Thermo U3000 system (Thermo Fisher Scientific, United States) with a YMC-Pack HPLC column (ODS-A C18 250 mm × 4.6 mm, 5 μm) and the column temperature was maintained at 40°C. The mobile phase consisted of methanol (A) and 0.1% formic acid (B). The injection volume was 20 μL, the flow rate of the mobile phase was 1 ml/min, and the UV and MS split ratio was 7:3. Analytes were eluted from the column in a gradient. The gradient procedure was carried out as follows: the initial composition of B was 93%, and after 40–50 min, it was reduced to 0%.

MS conditions: Analyses were conducted on a Thermo Q Exative spectrometer (Thermo Fisher Scientific, United States) in a positive mode combined with an electrospray ionization (ESI) source. The MS parameters were as follows: the sheath gas flow rate and aux gas flow rate were 40 L/h and 2 L/h, respectively. The capillary temperature and the aux gas heater temperature were set at 350 and 150°C, respectively. The spray voltage was set at 3.5 kV. Data were collected over the range of m/z 100–1,100 with a full ms dd ms2 mode.

### Xylene-Induced Ear Swelling Test

Six groups of 10 KM mice (male and female half) were treated with 0.5% CMC-Na (carboxymethyl cellulose sodium), QXWWD (680, 340, and 170 mg/kg), or aspirin (200 mg/kg). The QXWWD group’s dosage was calculated using its clinical dose (Heinrich M et al., 2020). The high, medium, and low dose groups of QXWWD were all dissolved in 0.5% CMC-Na. The drugs were given to the mice once a day for five days with an oral dose of 0.1 ml/10 g. On the sixth day, xylene (0.02 ml) was added to the posterior and anterior surfaces of the right ear. After 30 min, 0.5 ml of blood was collected from the orbit for white blood cell counts measurement. The mice were subjected to euthanasia while a 6-mm hole was punched out of both the treated and control ears. The ear swelling rate was expressed as the weight difference between the xylene treated ear (right ear, MR) and non-treated ear (left ear, ML). The formula is showed as following: (MR-ML)/ML.

### Antibacterial Activity Test

For antibacterial activity, the Kirby-Bauer (K-B) disc diffusion method was carried out on the agar plates. For control strains, three strains of *Staphylococcus aureus* (ATCC29213, ATCC25923, ATCC43300) with different drug resistance, *Enterococcus faecalis* (ATCC29212), *Streptococcus pneumoniae* (ATCC49619), *Pseudomonas aeruginosa* (ATCC27853), *Streptococcus pyogenes* (ATCC19615), *Klebsiella pneumoniae* (ATCC700603), *Escherichia coli* (ATCC25922), *Enterobacter cloacae* (ATCC700323) were used. A 20 μL sample solution was used for the impregnation of the filter discs of 6 mm. Also, a 6 mm diameter disc prepared with 20 μL normal saline was used as negative control while antimicrobials (Vancomycin: VA 30 μg, Imipenem: IPM 10 µg) were used as the positive controls. Antimicrobial activity was measured as a diameter of the inhibition zone around the disc after the incubation period of 24 h at 37°C, and the results were compared with negative and positive controls.

### Minimum Inhibitory Concentration (MIC) Determination

The samples were diluted into a series of concentration gradients (500, 250, 125, 62.5, 31.25, 15.625, 7.8125, and 3.90625 μg/ml) by the double dilution method. The samples were then added to the test tubes containing a 10 ml medium. Then, 50 μL of bacteria solution (1 × 10^5^ CFU/ml) was added to each test tube. All the tubes were cultured at 37°C for 24 h. The MIC was the lowest concentration of observable turbidity in the test tube that could be seen through the naked eye.

### Cell Culture and Viability Assay

The DMEM high glucose medium augmented with 10% (v/v) FBS, was employed for the growth of RAW 264.7 cells at 37°C and 5% CO_2_. For the evaluation of QXWWD's effect on cell viability, RAW264.7 cells were sown in 96-well plates for 24 h at a density of 5 × 10^3^ cells/well. For the treatment of these cells, different concentrations of QXWWD in the 0–500 μg/ml range were used for 24 h. Then, the culture medium was added with 10 µL of CCK-8 solution (100 µL/well) and was incubated at 37°C for 3 h. The absorbance was read with the help of a Multiskan MK3 microplate reader (Thermo Fisher Scientific, United States), at 450 nm and the IC_50_ (the half-maximal inhibitory concentration) was calculated using GraphPad Prism 8.0.

### Measurement of Pro-inflammatory Cytokines

RAW264.7 cells were transferred to the 96-well plates at a density of 5×10^3^ cells/well and incubated for 24 h. The cells were treated with different concentrations (80 μg/ml, 40 μg/ml, 20 μg/ml) of QXWWD for 24 h in the presence and absence of LPS (1 μg/ml). Murine TNF-α, IL-1, and IL-6 ELISA kits were used to measure pro-inflammatory cytokines TNF-α, IL-1, and IL-6 in cell-free supernatants according to the manufacturer’s instructions.

### Western Blot Analysis

RAW264.7 cells were cultured in high-glucose DMEM containing 10% FBS at 37°C in a incubator containing 5% CO_2_. To see how QXWWD affects these cells’ signaling pathways, cells were plated in 6-well plates (5 × 10^3^/well) and treated for 24 h with a variety of QXWWD concentrations (80 μg/ml, 40 μg/ml, 20 μg/ml), in the presence as well as in the absence of LPS (1 μg/ml). The cells were collected from the 6-well plates after being washed with ice-cold PBS followed by the addition of lysis buffer (Beyotime, Shanghai, China). The recovered lysate was incubated on ice for 30 min and centrifuged at 14,000 × g for 20 min at 4°C. Thereafter, the concentrations of protein were measured with a BCA Protein Assay Kit (Beyotime, Shanghai, China). An equal amount of protein (30 µg) was separated by 12% SDS-PAGE and transferred to the polyvinylidene difluoride (PVDF) membranes (Beyotime, Shanghai, China). The PVDF membranes were blocked with 5% fat-free milk and incubated with different primary antibodies against P38 (1:1,000 dilution), JNK (1:500 dilution), ERK (1:1,000 dilution), Akt (1:1,000 dilution), PI3K (1:1,000 dilution), P65 (1:1,000 dilution), p-P38 (1:1,000 dilution), p-JNK (1:1,000 dilution), p-ERK (1:1,000 dilution), p-Akt (1:1,000 dilution), p-PI3K (1:1,000 dilution), p-P65 (1:1,000 dilution), and β-actin/GAPDH (1:1,500 dilution) respectively for 18–24 h at 4°C and followed by washing twice. After incubation with the horseradish peroxidase-conjugated antibodies (1:1,000 dilution) for 0.5–1 h at room temperature, the target protein bands were detected with enhanced chemiluminescence substrate (Beyotime, Shanghai, China). Each WB analysis was performed three times. Semiquantitative analysis was performed using ImageJ software.

### Evaluation of Skin Irritation

Twelve rabbits were divided into two groups (normal skin group and damaged skin group), with six rabbits in each group. Before the experiment, hairs were removed from both sides of the experimental animals’ spines, with a hair-free area of 4 cm × 4 cm and an application area of 3 cm × 3 cm. To retain a little exudative in the skin, a sterilized surgical scalpel was used to gently scrape the damaged skin model. Then, 680 mg/kg of QXWWD was dissolved in 0.5% CMC-Na. For 7 days, 2 ml of QXWWD was applied topically on one side of the skin while 0.5% CMC-Na (2 ml) was applied topically on the other side. On daily basis, the medication site was observed for the presence of edema and erythema, which was noted 1, 24, 48, and 72 h after the last time drug, scored using skin irritation criteria, and assessed the stimulation intensity (Table 1).

**TABLE 1:** Standard scoring system for skin reactions.

Reaction score	Score
**Erythema**	
Absence of erythema	0
Very little erythema	1
Well established erythema	2
Moderate to severe erythema	3
Severe erythema (beet redness) to scar formation	4
**Edema**	
Absence of edema	0
Very little edema	1
Well established edema (edges of the area well defined by defined raising)	2
Moderate edema (raising approximately 1 mm)	3
Severe edema (raising more than 1 mm and extended beyond the area of exposure)	4
**The total possible score for primary irritation**	8
Absence of irritation	0–0.4
Very little irritation	0.5–1.9
Moderate irritation	2–4.9
Severe irritation	5–8

### Hematoxylin and Eosin (HE) Staining

The skin of the rabbit was peeled off about 1 cm^2^ in each experimental zone and was kept in paraformaldehyde (4%) for a duration of 1 h at 4°C. After dipping the tissues in 0.01 mol/L PBS for 5 min, they were dehydrated in an ethanol gradient. Following that, the tissues were vitrified with xylene and fixed in wax. The Leica RM2235 rotary microtome (Leica, Germany) was employed for the preparation of 5 μm thick sections. The slices were HE stained and were examined under a Leica DM2000 microscope (Leica, Germany).

### Statistical Analysis

All data were depicted as mean and standard deviations (SD) and compared the control and treated groups by one-way analysis of variance (ANOVA) or nonparametric Wilcoxon rank-sum test with Bonferroni corrections while using SPSS20.0, *p*-value <0.05 was considered statistically significant.

## Results

### Chemical Ingredients of QXWWD

In total, 1,058 chemical compounds were obtained from the TCMSP database, they were obtained from *Sophorae Flavescentis Radix* (490 compounds), *Fructus Cnidii* (222 compounds), *Artemisia Argyi Folium* (236 compounds), *Rhei Radix et Rhizoma* (110 compounds), and *Borneolum* (0 compounds).

### Network Construction and Analysis

**FIGURE 2:**
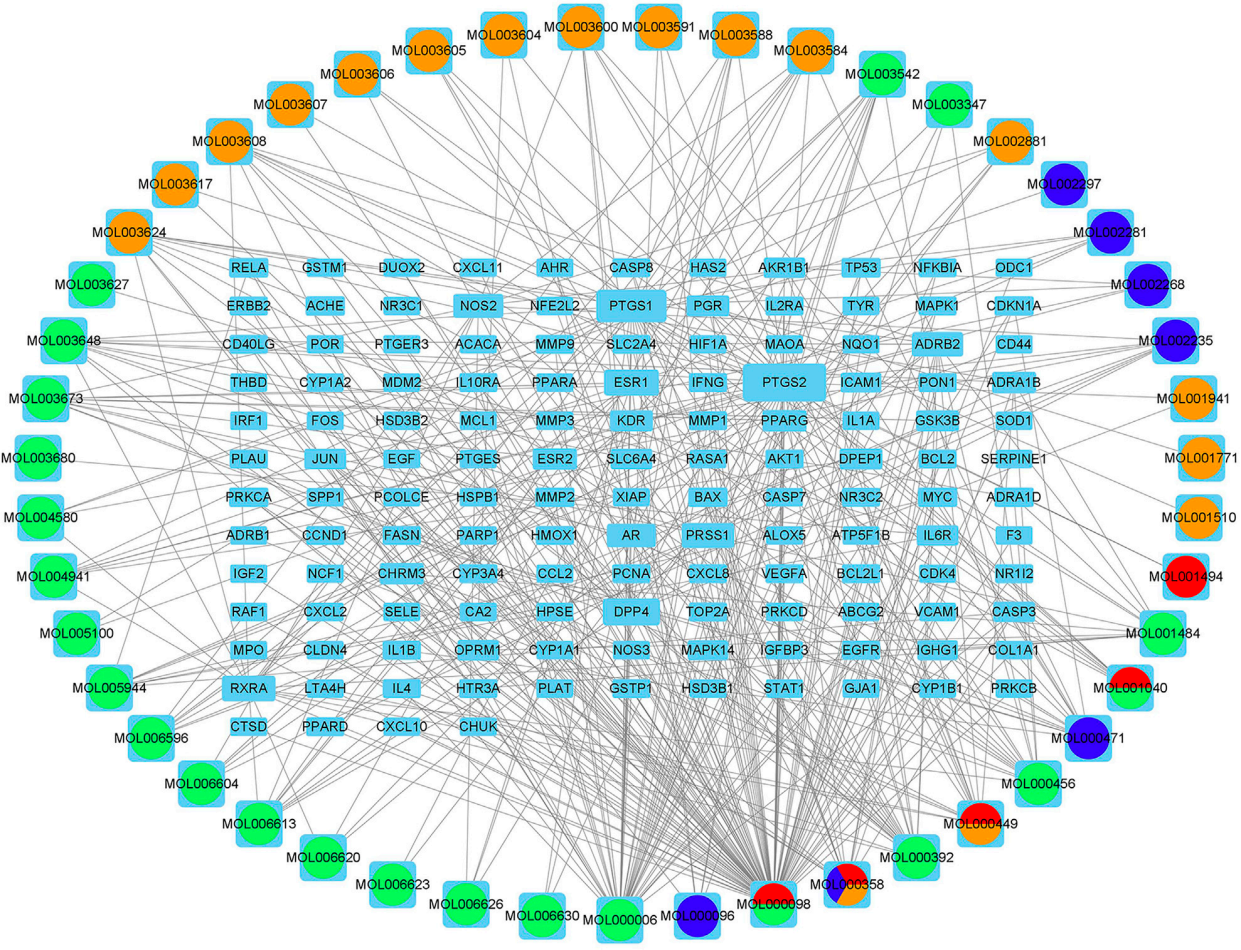
Compound-target network.

**FIGURE 3:**
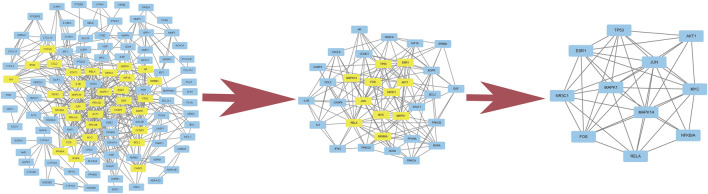
Hub genes of the network.

A total of 2,352 potential targets of disease were examined through “inflammation and bacteria”. These targets were added to the 1,058 targets of QXWWD to get a total of 136 core targets. The core targets were inputted into the network visualization software Cytoscape 3.8.0 to build the following network (Figure 2) which had 47 nodes. The larger node was of great significance. With regard to the degree analysis, the top ten compounds were quercetin, beta-sitosterol, stigmasterol, luteolin, formononetin, 8-Isopentenyl-kaempferol, aloe-emodin, phaseolin, *o*-Isovalerylcolum bianetin, and wighteone with 308-degree, 114-degree, 62-degree, 57-degree, 39-degree, 30-degree, 24-degree, 24-degree, 20-degree, and 19-degree respectively. Further details of these compounds have been depicted in Table 2. The targets (RELA, NFKBIA, MYC, MAPK1, MAPK14, ESR1, NR3C1, AKT1, TP53, JUN, and FOS) with a degree greater than the median were opted as the hub genes (Figure 3) and were considered for further molecular docking.

**TABLE 2:** The main chemical components of QXWWD.

Herb	Degree	Mol name	Main targets
*Artemisia argyi folium*, *Sophorae flavescentis radix*	308	Quercetin	PTGS1, PTGS2, RELA, EGFR, AKT1, FOS, JUN, IL6R, TP53, NFKBIA, MYC
*Artemisia argyi folium*, *Rhei Radix et Rhizoma*, *Fructus cnidii*	114	Beta-sitosterol	PTGS1, PTGS2
*Artemisia argyi folium*, *Fructus cnidii*	62	Stigmasterol	PTGS1, PTGS2
*Sophorae flavescentis radix*	57	Luteolin	PTGS1, PTGS2, RELA, EGFR, AKT1, MAPK1, JUN, IL6R, TP53, NFKBIA, IL4
*Sophorae flavescentis radix*	39	Formononetin	NOS2, PTGS1, MAPK14, ESR1, PKIA, JUN, IL4
*Sophorae flavescentis radix*	30	8-Isopentenyl-kaempferol	ESR1, PTGS2, MAPK14
*Rhei Radix et Rhizoma*	24	Aloe-emodin	PTGS1, PTGS2, PKIA, TP53, MYC, IL1B
*Sophorae flavescentis radix*	24	Phaseolin	PTGS1, ESR1, PTGS2, MAPK14
*Sophorae flavescentis radix*	20	*o*-Isovalerylcolum bianetin	PTGS1, PTGS2
*Sophorae flavescentis radix*	19	Wighteone	ESR1, PTGS2, MAPK14

### Biological Function and Pathway Analysis

**FIGURE 4:**
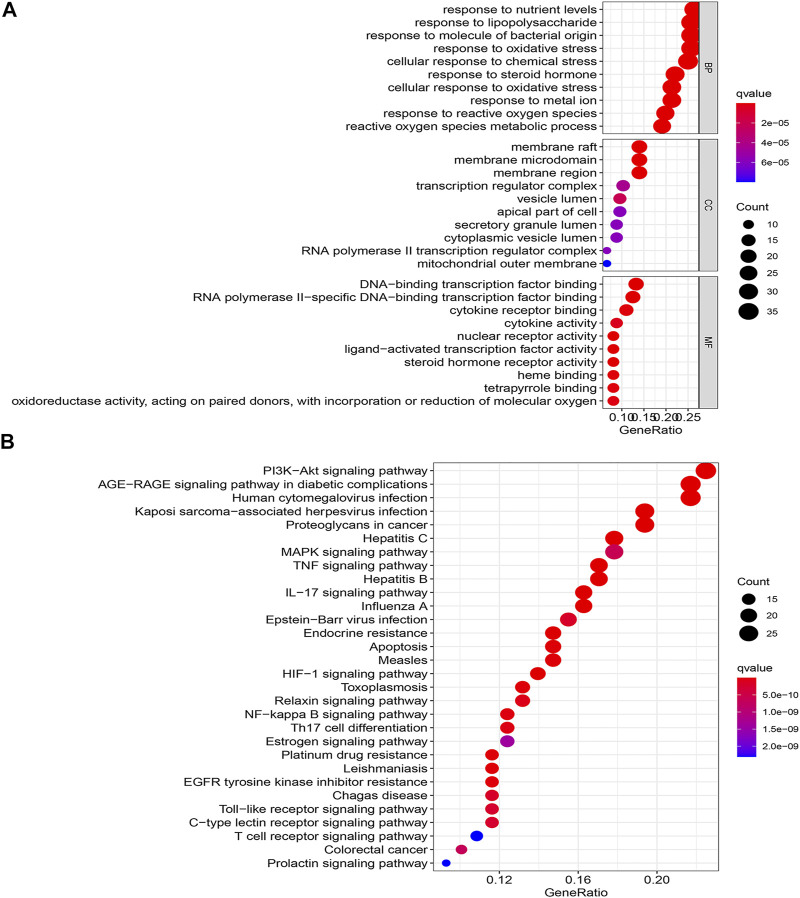
GO and KEGG enrichment analyze **(A)**: GO enrichment analyze, **(B)**: KEGG enrichment analyze.

To deeply explore the underlying mechanism of drug treatment, GO and KEGG enrichment analyses were conducted with the R platform on 136 core targets. A total of 2088 terminologies related to biological events were chosen, specifically included inflammatory response, response to bacteria, response to nutritional level, response to lipopolysaccharide, response to oxidative stress, etc. (Figure 4A). Concerning the enrichment analysis of cellular components, the targets were comprised of the cell membrane. Concurrently, the molecular function terms primarily comprised steroid hormone receptor activity, ligand−activated transcription factor activity, nuclear receptor activity, and so on. The main effects linked to the QXWWD were grouped using the KEGG pathway enrichment analysis. A sum of 30 top-ordered pathways (Figure 4B) were screened out (*p* < 0.05). Among them, the most important pathways included the MAPK signaling pathway, TNF signaling pathway, PI3K-Akt signaling pathway, AGE-RAGE signaling pathway, and other pathways associated with immunity and inflammation.

### Molecular Docking of Main Chemical Compounds Binding to Hub Genes

**FIGURE 5:**
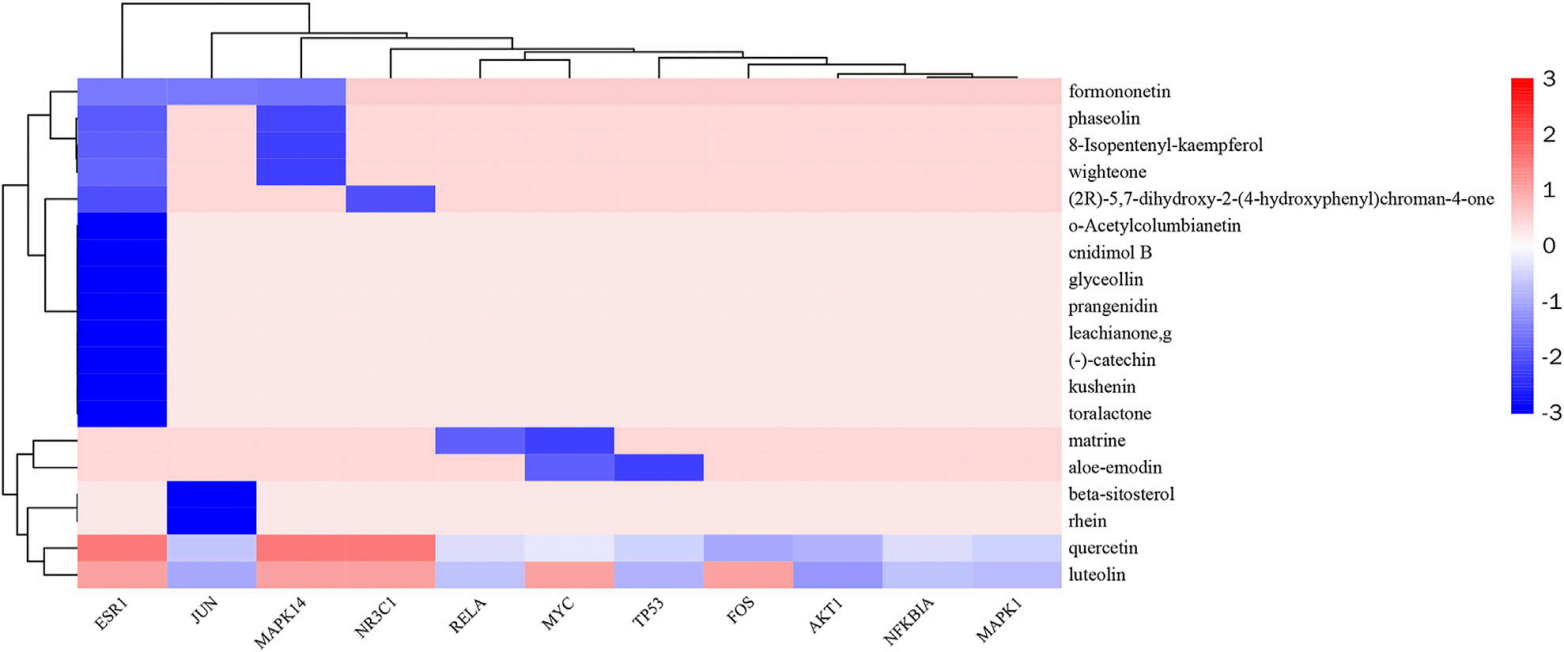
Heatmap of docking affinity of hub genes.

**FIGURE 6:**
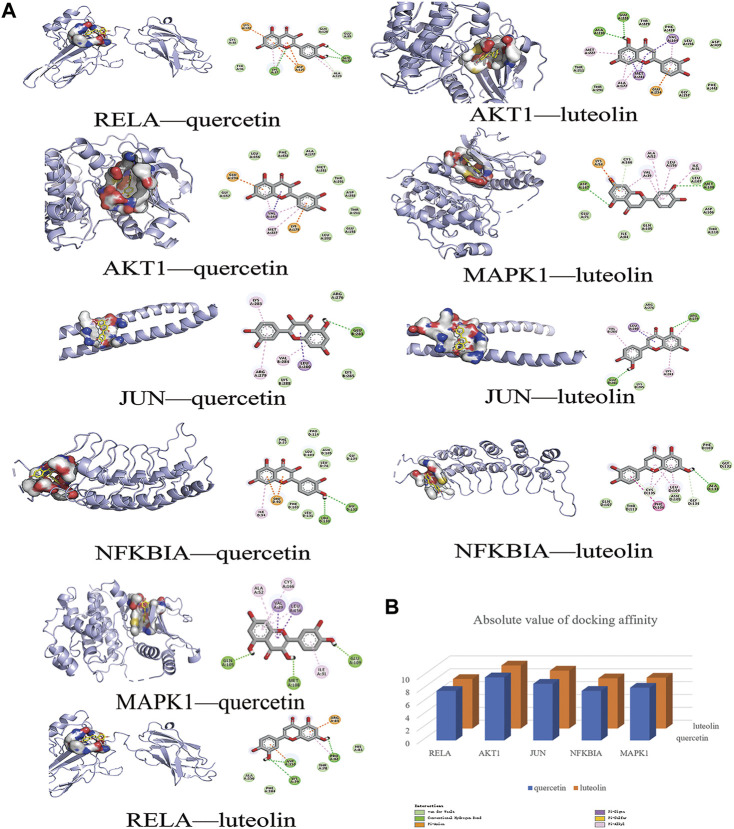
Molecular docking models of main chemical compounds binding to potential targets. **(A)** Molecular docking models of main chemical compounds, **(B)** Absolute value of docking affinity.

In this study, the most expected interaction activity between 11 hub genes and their corresponding compounds of QXWWD was assessed by using molecular docking verification (Figure 5). However, the main chemical compounds, as well as the targets, were filtered using AutoDock Vina’s docking affinity values. Thus, the larger the absolute value of the docking affinity, the greater the binding capability between the active sites of targets and compounds. For concreteness, quercetin and luteolin were selected for molecular docking with the hub genes (Figure 6).

### Thin Layer Chromatography

**FIGURE 7:**
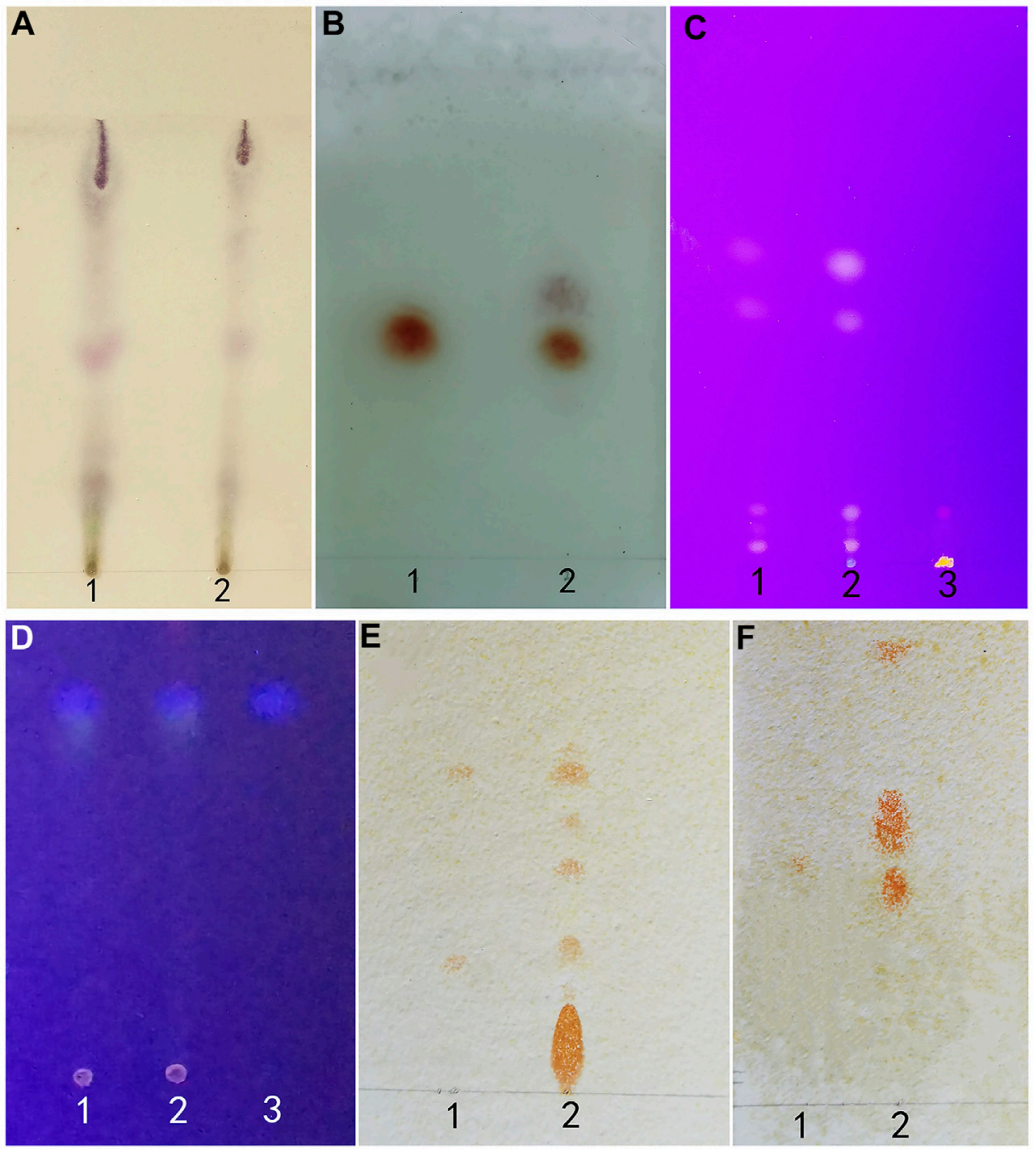
**(A)** (*Artemisia Argyi Folium*): 1 test herb 2 control herb. **(B)** (*Borneolum*): standard herb 2 test herb. **(C)** (*Rhei Radix et Rhizoma*): 1 control herb 2 test herb 3 standard herb. **(D)** (*Fructus Cnidii*): 1 standard herb 2 test herb 3 control herb. **(E)** (*Sophorae Flavescentis Radix*): 1 matrine and sophoridine 2 test herb. **(F)** (*Sophorae Flavescentis Radix*): 1 oxymatrine 2 test herb.

Within the chromatogram of the sample solution, the same color spots were represented in corresponding positions with standard herb or control herb solution (Figure 7).

### The Main Chemical Components in QXWWD

**FIGURE 8:**
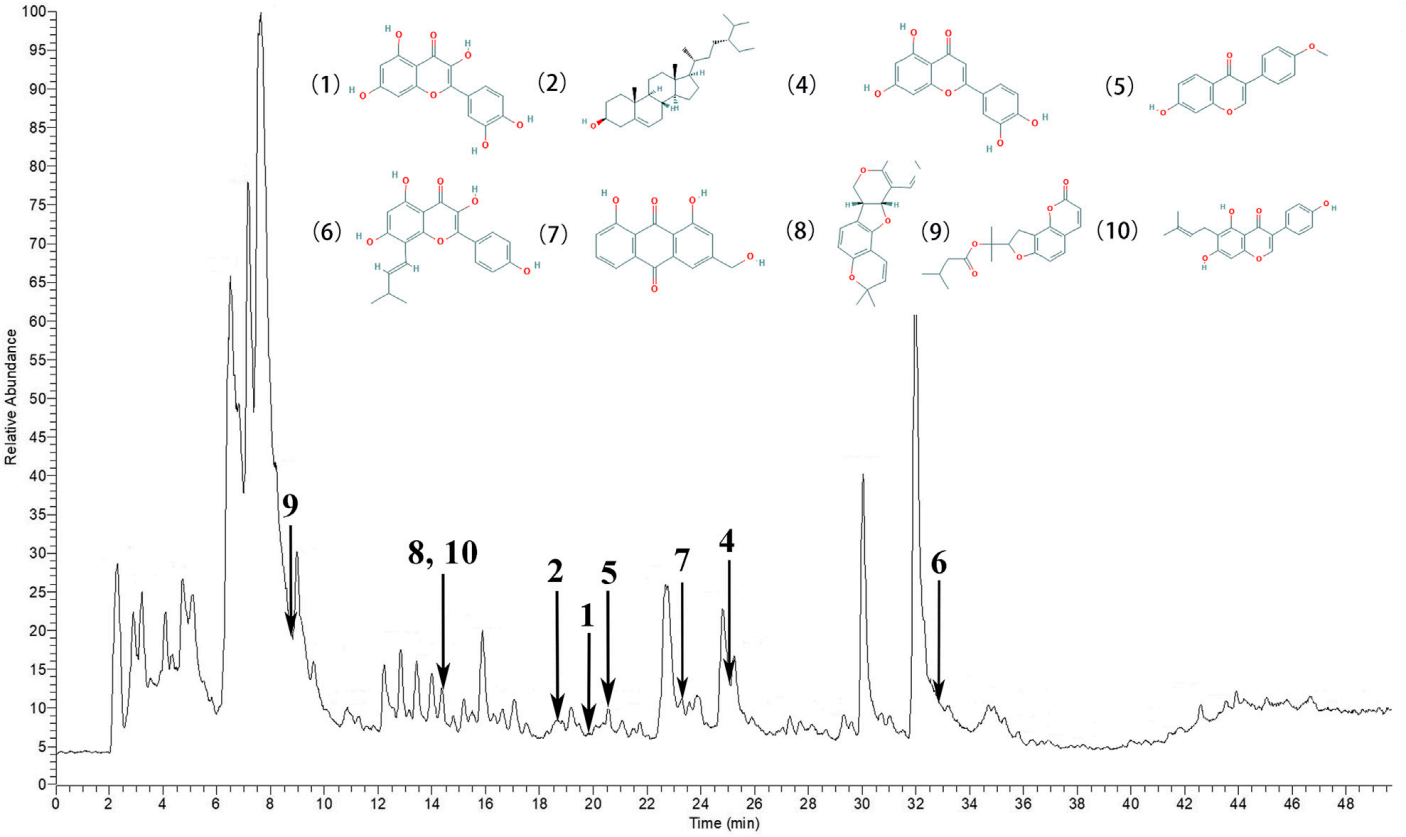
The peaks in the Q Exative chromatogram. The Q Exactive MS of the QXWWD (1) quercetin, (2) beta-sitosterol, (4) luteolin, (5) formononetin, (6) 8-Isopentenyl-kaempferol, (7) aloe-emodin, (8) phaseolin, (9) *o*-Isovalerylcolum bianetin, (10) wighteone.

According to the degree analysis and HPLC-Q-Exactive-MS, from the QXWWD, among the top ten compounds (Table 2), nine compounds including quercetin, beta-sitosterol, luteolin, formononetin, 8-Isopentenyl-kaempferol, aloe-emodin, phaseolin, *o*-Isovalerylcolum bianetin and wighteone were obtained. The peaks in the Q Exative chromatogram (Figure 8 and Supplementary Figures S1–S9) confirmed the presence of these compounds.

### Anti-Inflammatory Effects of QXWWD in the Animal Model

**FIGURE 9:**
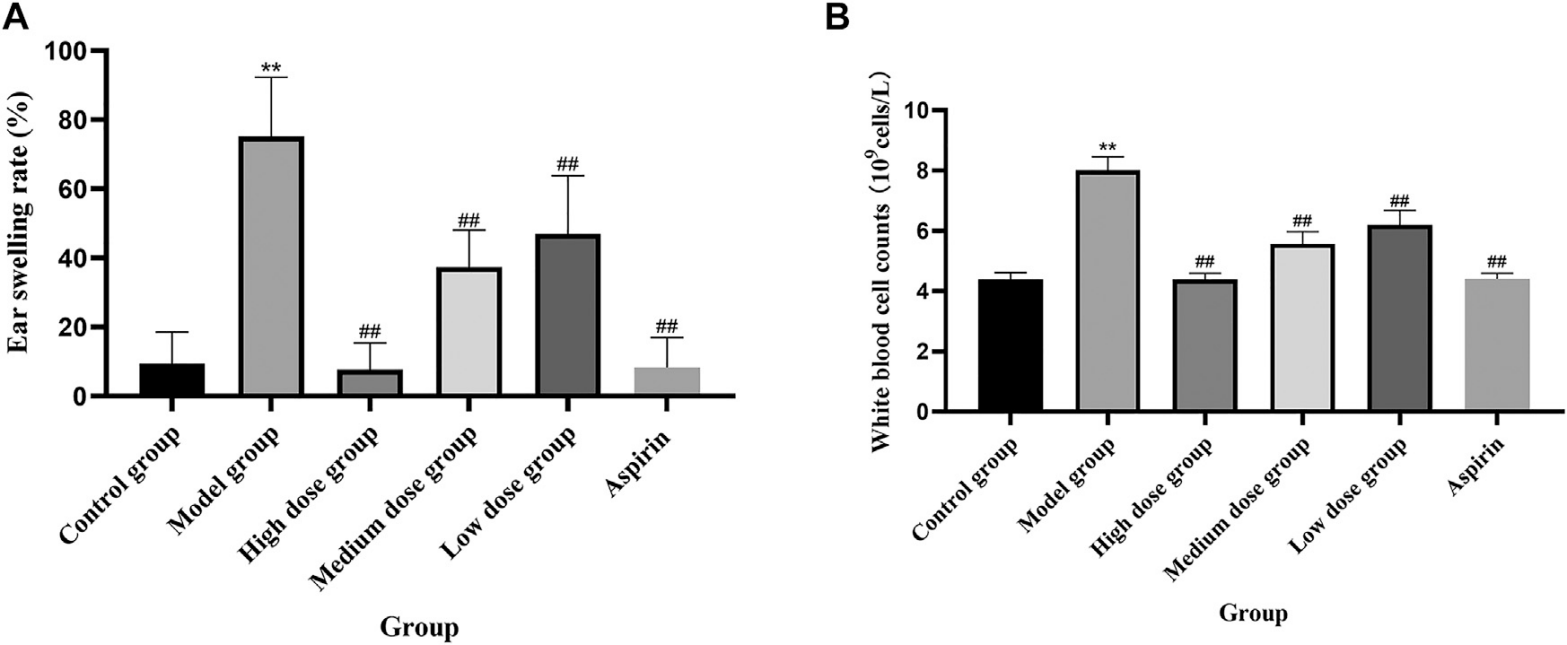
Xylene-induced ear swelling in mice. **(A)** Ear swelling rate, **(B)** White blood cell counts.

The ear swelling and the white blood cell (WBC) counts of the model group were considerably greater (**p < 0.01) in contrast with the control group. In contrast with the model group, each dose level group of QXWWD depicted spectacular suppressing effects on xylene-driven ear swelling and the white blood cell counts in a concentration-dependent manner (^##^ *p* < 0.01 compared with the model group). The results are presented in Figure 9.

### Antibacterial Activities of QXWWD

**FIGURE 10:**
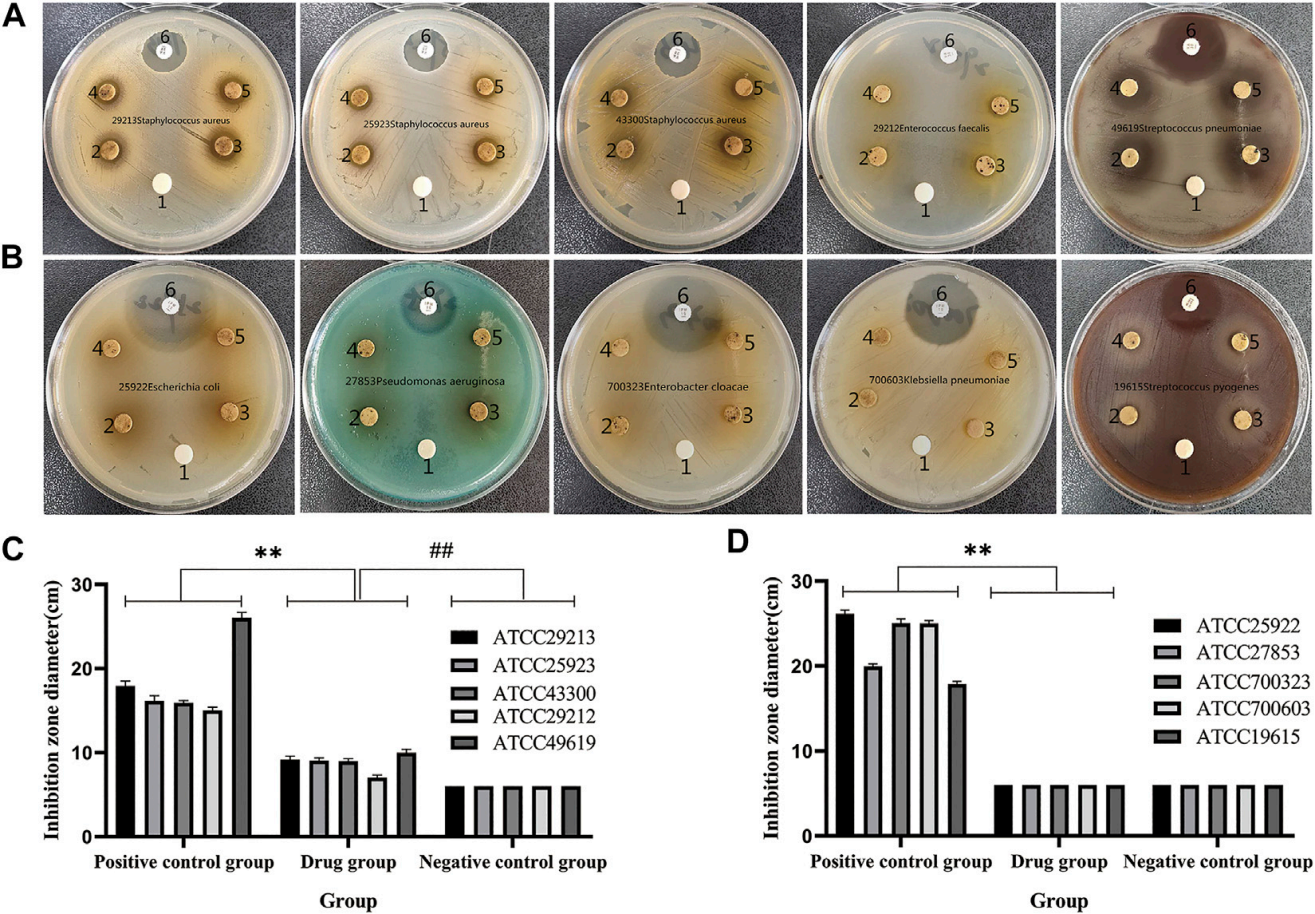
Antibacterial activity test. **(A and C)** QXWWD had a significant inhibitory effect on these bacteria, **(B and D)** QXWWD had no obvious inhibitory effect on these bacteria.

QXWWD was tested for its antibacterial activity. As shown in Figure 10, position 1 in the agar plates was the negative control, positions 2, 3, 4, and 5 were the drug groups, and position 6 was a positive control. In contrast with the negative control group, QXWWD had a significant inhibitory effect on *Enterococcus faecalis* (ATCC29212), *Staphylococcus aureus* (ATCC29213, ATCC25923, ATCC43300), and *Streptococcus pneumoniae* (ATCC49619) but had no obvious inhibitory effect on *Streptococcus pyogenes* (ATCC19615), *Enterobacter cloacae* (ATCC700323), *Escherichia coli* (ATCC25922), *Pseudomonas aeruginosa* (ATCC27853), and *Klebsiella pneumoniae* (ATCC700603). (**p < 0.01 in contrast with the positive control group, ^##^ *p* < 0.01 in contrast with the negative control group). Besides, MICs of QXWWD were found to be 7.8125 μg/ml, 7.8125 μg/ml, 7.8125 μg/ml, 15.625 μg/ml and 250 μg/ml against *Staphylococcus aureus* (ATCC29213, ATCC25923, ATCC43300), *Streptococcus pneumoniae* (ATCC49619) and *Enterococcus faecalis* (ATCC29212) respectively (Supplementary Figures S10–S14).

### QXWWD Inhibited the Expression of Multiple Inflammatory Cytokines in RAW264.7 Cells

**FIGURE 11:**
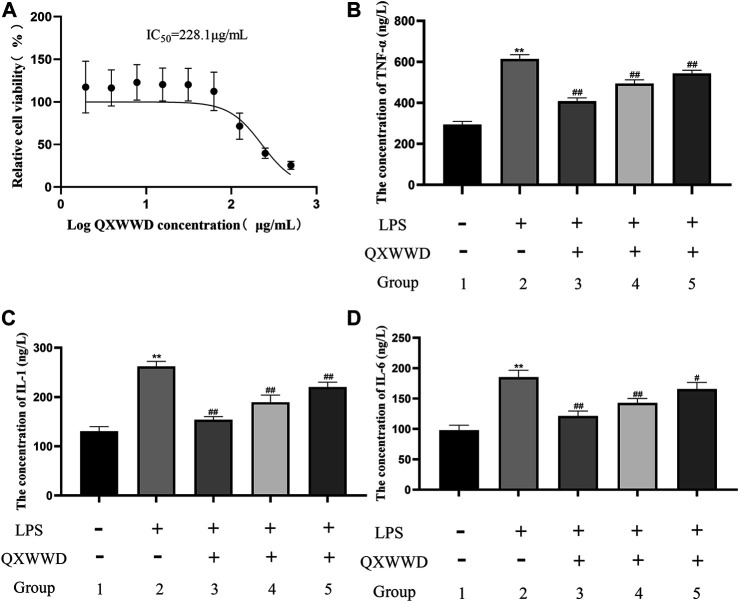
Cell viability assay and measurement of pro-inflammatory cytokines 1: control group; 2: model group (1 μg/ml LPS); 3: high dose group (80 μg/ml QXWWD+1 μg/ml LPS); 4: medium dose group (40 μg/ml QXWWD+1 μg/ml LPS); 5: low dose group (20 μg/ml QXWWD+1 μg/ml LPS). **(A)** Relative cell viability, **(B)** The concentration of TNF-α in cell-free supernatants, **(C)** The concentration of IL-1 in cell-free supernatants, **(D)** The concentration of IL-6 in cell-free supernatants.

As shown in Figure 11A, IC_50_ was found to be 228.1 μg/ml when using the Graph Pad Prism 8.0. Compared with the control group, LPS could significantly induce RAW 274.7 cells to secrete inflammatory cytokines, such as TNF-α, IL-1 and IL-6 (^**^ *p <* 0.01 compared with the control group). In comparison to the model group, 20–80 μg/ml of QXWWD in LPS-effectuated RAW 264.7 cells greatly suppressed the release of inflammatory mediators such as IL-1, IL-6 and TNF-α (^#^ *p<* 0.05, ^##^ *p<* 0.01 compared with the model group) in a dose-dependent manner (Figures 11B, C, D).

### QXWWD Regulated the PI3K-Akt and MAPK Signaling Pathways in RAW264.7 Cells

**FIGURE 12:**
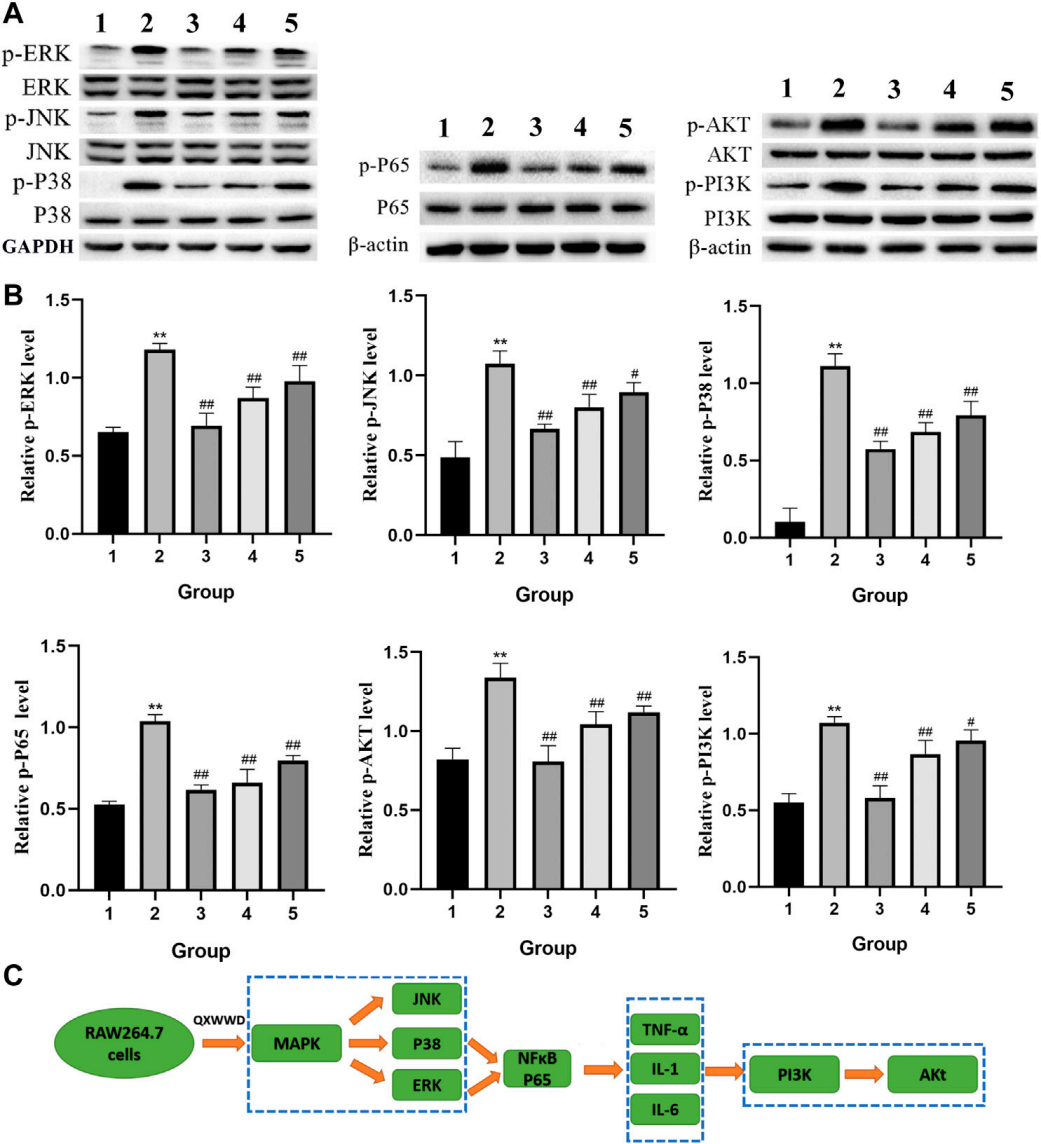
QXWWD treatment regulates the expression of key nodes in PI3K-Akt and MAPK signaling pathways activated in RAW264.7 cells 1: control group; 2: model group (1 μg/ml LPS); 3: high dose group (80 μg/ml QXWWD+1 μg/ml LPS); 4: medium dose group (40 μg/ml QXWWD+1 μg/ml LPS); 5: low dose group (20 μg/ml QXWWD+1 μg/ml LPS). **(A)** Representative bands of protein levels detected by WB, **(B)** The relative protein levels, **(C)** QXWWD regulated the PI3K-Akt and MAPK signaling pathways in RAW264.7 cells.

Based on the importance of “top 10” KEGG pathways analyzed and the hub genes of the network, PI3K-Akt, MAPK signaling pathways as well as nuclear factor NF-κB P65 were selected for experimental validation as major bacterial and inflammation-related signaling pathways. As shown in Figure 12A, B, after 24 h treatment with different concentrations of QXWWD (80 μg/ml, 40 μg/ml, 20 μg/ml), the phosphorylated protein levels of P38, JNK, ERK, Akt, PI3K, and P65 were significantly decreased in a dose-dependent manner (^**^ *p <* 0.01 compared with the control group and ^#^ *p <* 0.05, ^##^ *p <* 0.01 compared with the model group). Taken together with the results of pro-inflammatory cytokines measurement, the anti-inflammatory and antibacterial activities of QXWWD seem to be mediated by inhibition of the exaggerated release of inflammatory cytokines including IL-1, IL-6, TNF-α as well as regulation of P65, MAPK and PI3K-Akt signaling pathways (Figure 12C).

### The Possible Therapeutic Role of External Usage of QXWWD

**FIGURE 13:**
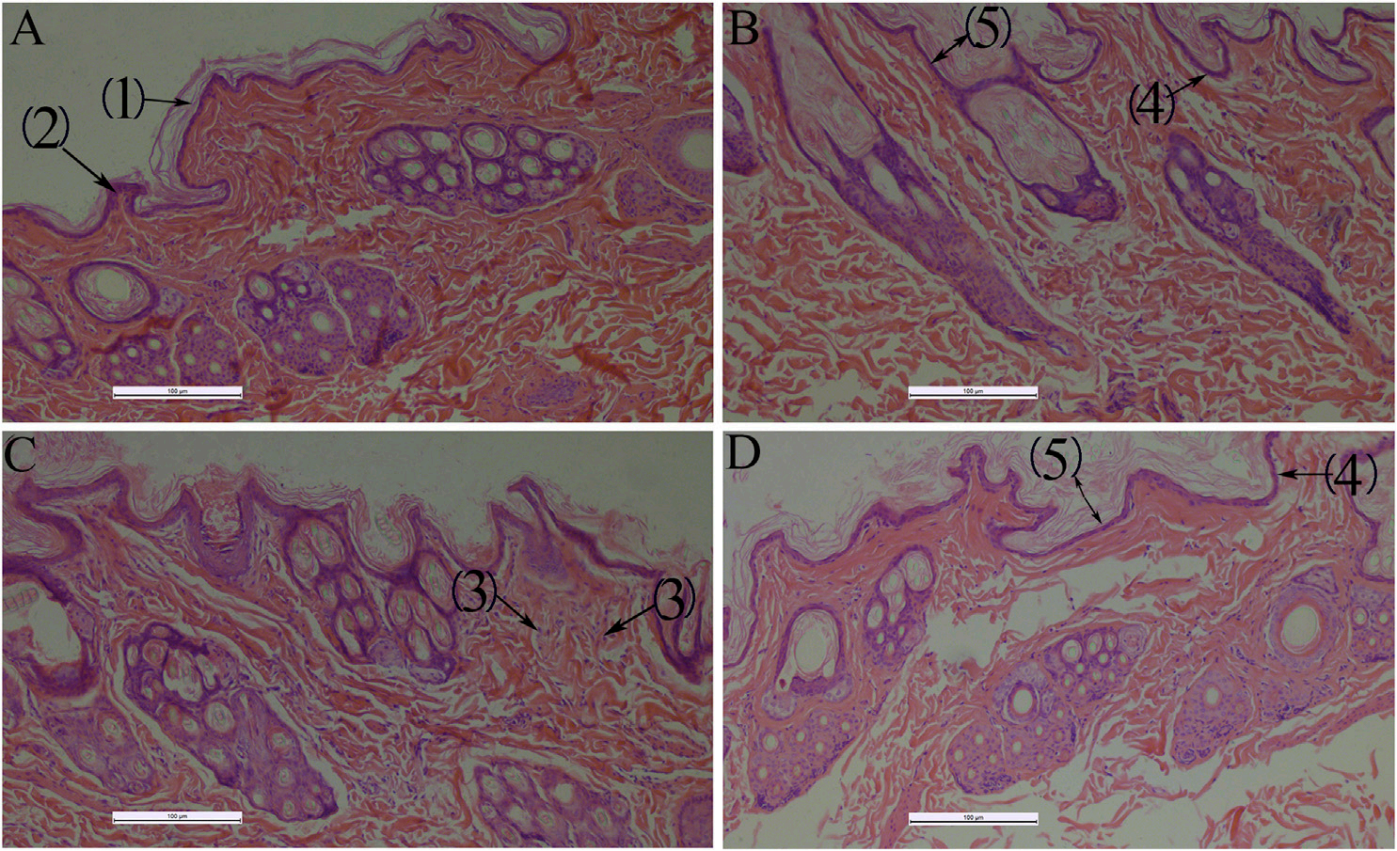
Histological photomicrographs of a frozen section taken from rabbit treated with several applications 100×, **(A)**: Normal skin-control **(B)**: Normal skin-drug, **(C)**: Damaged skin-control, **(D)**: Damaged skin-drug; (1): Stratum corneum, (2): Stratum spinosum, (3): Inflammatory cells, (4): Stratum basale, (5): Epidermis.

QXWWD did not irritate healthy skin, but it did irritate damaged skin mildly (Supplementary Tables S7–S11). During the microscopic examination, HE staining revealed that the skin structure of control groups (Figure 13) was normal and the epidermal layers were firmly and neatly arranged with clear stratification. The stratum corneum cells were loosely organized after using QXWWD, and the intercellular space in the spinous layer increased. The results revealed that QXWWD’s increased permeability was linked to changes in skin microstructure. For the damaged skin drug group, the decreased inflammatory cells under the stratum spinosum also demonstrated the therapeutic effects of QXWWD relative to the damaged skin control group.

## Discussion

QXWWD is a Chinese folk prescription for the treatment of inflammatory and bacterial diseases that has a long history of clinical practice. QXWWD has a wide range of applications, including the prevention and treatment of traumatic infections, as well as the clinical treatment of acute and chronic dermatitis and other common skin diseases. Our findings revealed that QXWWD ethanolic extract has good antibacterial (inhibition of *Enterococcus faecalis*, *Staphylococcus aureus,* and *Streptococcus pneumoniae*) and anti-inflammatory (suppressing the ear swelling, elevated white blood cell counts, and the levels of TNF-α, IL-1, and IL-6) effects *in vitro*. The transcutaneous mechanism of QXWWD, which was tested using skin irritation and HE staining, revealed the drug's potential therapeutic function by disrupting the cuticle's microstructure. Based on the network pharmacology and molecular docking, JUN, MAPK1, RELA, NFKBIA, MYC, and AKT1 were found to be the potential identified key targets. The effects of QXWWD on these targets were further verified by western blot. The MAPK and PI3K-Akt signaling pathways were found to be among the top 10 KEGG pathways, with the genes encoding them serving as treatment targets. Both the PI3K-Akt and MAPK signaling pathways were chosen for experimental validation due to lower *p* values and more key targets identified through KEGG analysis. For the PI3K-Akt signaling pathway, it mediates the proliferation, apoptosis, differentiation, and migration in various cells. Our findings show that QXWWD can reduce the levels of phosphorylated Akt protein in RAW264.7 cells, indicating that it can play a role in the treatment of inflammatory and bacterial diseases by controlling relevant signaling pathways. MAPK signaling pathway also plays a crucial role in inflammation and infection. Particularly, NF-κB is a key factor in regulating inflammatory mediators during the process of inflammation. Drug stimulation of nuclear factor NF-κB increased the levels of pro-inflammatory cytokines such as TNF-α, IL-1, and IL-6. Phosphorylated protein expressions of key targets in the MAPK pathway were significantly decreased after QXWWD treatment. Besides, protein expression of NF-κB p-P65 was also decreased. Current data elucidated that QXWWD could exert better anti-inflammatory and antibacterial effects *via* blocking the MAPK pathway.

According to network pharmacological analysis, we examined ten important chemical components of QXWWD: quercetin, beta-sitosterol, stigmasterol, luteolin, formononetin, 8-Isopentenyl-kaempferol, aloe-emodin, phaseolin, *o*-Isovalerylcolum bianetin, and wighteone. Among the top ten compounds, nine compounds including quercetin, beta-sitosterol, luteolin, formononetin, 8-Isopentenyl-kaempferol, aloe-emodin, phaseolin, *o*-Isovalerylcolum bianetin, and wighteone were obtained by the HPLC-Q-Exactive-MS. Previously, the anti-inflammatory, as well as the immunomodulation properties of quercetin and luteolin, have been extensively implicated in various inflammatory incidents, suggesting that QXWWD may probably play a significant role in the suppression of inflammatory diseased conditions characterized by the immunoinflammatory response. In many investigations, the researchers have found that luteolin arrested the association between p65 (RELA) and transcriptional coactivator and also suppressed the NF-κB transcriptional activity in Rat-1 fibroblast stimulated with LPS (Che et al., 2020). Also, luteolin was found to affect the MAPK pathway and thereby inhibited the IL-1β-induced c-Jun N-terminal kinase (JNK) and p38 kinase stimulation in SW982 cells (Choi and Lee, 2010). Quercetin, one of the main components of QXWWD, was found to inhibit the LPS activated inflammatory responses in mononuclear cells by arresting the TLR2/NF-κB signaling pathways that serve as a connecting bridge between specific and non-specific immunity and could be a useful candidate for the management of inflammatory conditions involving factors like PI3K, NF-κB and TNFα (Zhang et al., 2016). Considering the importance of the two compounds, based on the authentic compound standards (≥99% purity), quercetin and luteolin were identified in our QXWWD. The HPLC chromtograms of quercetin and luteolin were presented in Supplementary Figure S15. Furthermore, to deeply understand the underlying mechanism of QXWWD in the cure of inflammatory disease, the 136 targets were examined for 11 hub genes in the PPI network. Unexpectedly, the PPI network contained six targets (e.g., RELA, AKT1, NFKBIA, MYC, JUN, and MAPK1), which are considered to be the most important genes involved in the 11 hub genes, reconfirming QXWWD might possess good effects against inflammatory disease. Also, we conducted the simulation of molecular docking between six hub genes and two main chemical compounds to complement main targets. The obtained results indicated good docking affinities for all of the pairs of target compounds. Moreover, the pro-inflammatory genes such as RELA, AKT1, JUN, NFKBIA, MYC, and MAPK1 have confirmed the involvement in the onset of inflammatory diseases, particularly, in terms of immune inflammation. The commensal organisms such as anaerobic gut bacteria have been found to regulate the shuttling of RELA and PPAR-gamma between cytoplasm and nucleus and thus play a key role in an inflammatory condition (Kelly et al., 2004). The study reports that AKT1/NF-κB signaling pathways are involved in the production of IL-6, which is widely implicated in the pathogenesis of autoimmune disorders (Zhang et al., 2013). The elevated levels of serum TNF during the inflammatory response were mediated by AKT1/NF-κB signaling pathways, as previously stated (Zhang et al., 2013). Recently, studies demonstrated that c-Jun N-terminal kinase 2 (c-JUN) knockout mice with passive murine collagen lead to Joint damage and inflammation (Han et al., 2002). Besides this, Gene variants in NFKBIA facilitate inflammation and display a crucial role in the event of atherosclerosis and coronary artery disorders (Coto et al., 2019). A study showed that c-Myc is considerably associated with several cellular events including cell proliferation, differentiation, and growth. The c-Myc was found to upregulate the TNF-α, TGF-β, IL-8, and IL-10 whereas, TNF-α, TGF-β, IL-1, IL-2, and IL-4 enhanced c-Myc expression (Nakashima et al., 2005). These interactions depicted a key role in different inflammatory conditions. MAPK1 is involved in several signaling pathways that control a variety of cellular events such as apoptosis and proliferation (Zabihula et al., 2019). As a result, the study's findings revealed that RELA, AKT1, JUN, NFKBIA, MYC, and MAPK1 play an important role in apoptosis, proliferation, and immune inflammation responses and that inhibiting these processes may be an effective therapeutic target for QXWWD against inflammatory disease. The results were further verified by molecular docking. The results revealed that luteolin displayed a strong affinity toward RELA, AKT1, JUN, NFKBIA, and MAPK1, while luteolin had a strong affinity for RELA, AKT1, JUN, NFKBIA, and MYC. As a result, quercetin and luteolin can play a key role in apoptosis, antioxidant activity, angiogenesis, cell cycle, and inflammation. The KEGG enrichment analysis revealed that the main targets were abundantly located in certain signaling pathways like PI3K/AKT, MAPK, TNF, and NF-κB signaling pathways. The findings of the PPI network indicated that RELA, NFKBIA, MYC, MAPK1, MAPK14, ESR1, NR3C1, AKT1, TP53, JUN, and FOS were the possible hub genes. In light of these findings, we hypothesize that QXWWD may regulate inflammatory disease *via* the following pathways. The PI3K/AKT signaling pathway is mainly attributed to regulate the stimulation of inflammatory response cells and also the release of inflammatory transmitters to perform an important role during the chronic inflammatory response (Zhang et al., 2018). Likewise, the MAPK signaling pathway is responsible for transmitting signals from the cellular membrane toward the nucleus in response to various stimuli. This pathway regulates a broader spectrum of cellular events such as growth, stress responses, and inflammation, thus, it is a broadly implicated therapeutic target for tumor and peripheral inflammatory conditions (Yuan et al., 2017). TNF is a well-known pleiotropic cytokine and has been demonstrated with several important roles in disease pathogenesis as well as homeostasis. The TNF receptor signaling model has also been expanded to include the development of specific signaling complexes, which are commonly linked to functional outcomes such as necroptosis, apoptosis, and inflammation (Borghi et al., 2016). It is worthwhile to mention the role of transcriptional factors belonging to the NF-κB family, which play significant roles in innate immunity and inflammation as well (Sun, 2017). Also, NF-κB is a widely acknowledged key player in several steps linked with the onset and subsequent progression of inflammation (Karin and Greten, 2005). The NF-κB has also been found to facilitate several other signaling pathways and molecules during such a process.

In many instances, inflammation has been appeared to be one of the most complex processes, responsible for several events including tissue breakdown and repair, enzyme activation, cell migration, pro-inflammatory cytokines release, and extravasation of fluid (Castello et al., 2017). As a result, when conducting pharmacological studies, the use of specific experimental models becomes important. Acute inflammation is characterized by the formation of redness, proliferative cells, swelling, heat, and pain (Habtezion, 2015). Therefore, swelling and exudation of fluid are important manifestations of acute inflammation (Ruiz et al., 2019). Xylene can cause ear swelling in mice which leads to fluid accumulation, elevated white blood cell counts and triggers an acute inflammatory response (Zeng et al., 2020). In contrast, chronic inflammation is characterized by the formation of proliferative cells, which occurs when the acute response fails to kill proinflammatory agents (Zhao et al., 2016). In our study, QXWWD was found to significantly decrease ear swelling and white blood cell counts in mice. Thus, the suppression of the said response is quite indicative of the antiphlogistic effect. LPS is one of the key mediators responsible for tissue injuries, which is frequently originating from the infection of Gram-negative bacterial consortia (Cheng et al., 2018). TNF-α is a classic inflammation indicator, which can initiate the production of various inflammatory cytokines including IL-1and IL-6 (Kauppinen et al., 2013). Compared with the model group, 20–80 μg/ml of QXWWD in LPS-induced RAW 264.7 cells greatly inhibited the release of inflammatory mediators, mainly of IL-1, Il-6, and TNF-α in a dose-dependent manner, suggesting that QXWWD was effective in inflammatory conditions.

Bacterial infection is one of the most important causes of inflammation. Recently, knockout mouse models were used to reveal the consequences of pro-inflammatory and tumorigenic properties of the colon’s normal bacterial flora (Yang and Pei., 2006). Therefore, we conducted an antibacterial experiment to evaluate the antibacterial properties of QXWWD. Antibacterial experiments found that QXWWD had inhibitory effects on pathogenic bacteria of *Enterococcus faecalis* (ATCC29212) (MIC = 250 μg/ml), *Staphylococcus aureus* (ATCC29213, ATCC25923, ATCC43300) (MIC = 7.8125 μg/ml), *Streptococcus pneumoniae* (ATCC49619) (MIC = 15.625 μg/ml) but had no obvious inhibitory effect on *Streptococcus pyogenes* (ATCC19615), *Enterobacter cloacae* (ATCC700323), *Klebsiella pneumoniae* (ATCC700603), *Escherichia coli* (ATCC25922), and *Pseudomonas aeruginosa* (ATCC27853). *Staphylococcus aureus* is most frequently isolated from the skin microbiota of dermatitis patients (Geoghegan et al., 2018). Due to *Staphylococcus aureus* infection, the diversity of the microorganisms is reduced as well as the normal microbiota on the skin is disrupted (Geoghegan et al., 2018). Thus, drugs that inhibiting *Staphylococcus aureus* with a low MIC can play a good role in the treatment of acute and chronic dermatitis. These results were consistent with the good therapeutic effects of QXWWD in our previous clinical applications. *Enterococcus faecalis*, one of the most frequently isolated bacterial species in animal wounds, is an anaerobic Gram-positive bacterial that normally commences in the human oral cavity, gastrointestinal tract, and vagina because it has demonstrated good adaptation to such environments with rich nutrient and low oxygen levels (Chong et al., 2017; Alghamdi and Shakir, 2020). *Enterococcus faecalis* is the most frequently detected bacteria in cases of post-endodontic therapy pain and infection, with prevalence values as high as 90% (Alghamdi and Shakir, 2020). *Streptococcus pneumoniae* colonizes the human nasopharynx and can cause a variety of diseases, including otitis media, pneumonia, bacteraemia, and meningitis (Mitchell and Mitchell, 2010). The results of antibacterial experiments directly demonstrated that the potential mechanism of the Chinese herbal formula QXWWD in the treatment of bacterial, acute, and chronic dermatitis and provide additional evidence for the promotion of the wide use of QXWWD in the clinic for the treatment of diseases such as periodontitis and bacterial pneumonia.

The comprehensiveness, systematicness and integrity of network pharmacology are in line with the characteristics of multi compound, multi-target and multi-channel of Chinese herbal medicine, and it is expected to become an important tool in the research of traditional Chinese medicine. Despite its broad application prospects, network pharmacology still has some limitations. Firstly, the incomplete existing database will lead to the lack of analysis data. Secondly, the current network pharmacology technology is difficult to achieve the goal of quantification. Finally, most studies based on network pharmacology are still static network analysis. Therefore, a large number of *in vivo* or *in vitro* experiments are needed. In the future, with the popularization of network pharmacology technology, an ultra high throughput, fast and non-destructive method can be found to effectively solve the above limitations.

## Conclusions

Based on the network pharmacology and molecular docking analysis, the underlying mechanism of QXWWD in dermatitis therapy involves the regulation of pathways and targets in multiple biological processes. The antibacterial and anti-inflammatory effects of QXWWD in bacteria, cells, and animal models seem to be mediated by inhibition of the release of pro-inflammatory cytokines including TNF-α, IL-1, IL-6, and regulation of NF-κB, ERK, P38 MAPK, and PI3K-Akt signaling pathways. Overall, the obtained results suggested that QXWWD might be used as a promising therapeutic agent for bacterial, acute, and chronic dermatitis and provide additional evidence for the promotion of the wide use of QXWWD in the clinic for the treatment of diseases such as periodontitis and bacterial pneumonia.

## Data Availability

The raw data supporting the conclusions of this article will be made available by the authors, without undue reservation, to any qualified researcher.
