# Supplementary material for: Mechanism of Anti-Inflammatory and Antibacterial Effects of QingXiaoWuWei Decoction Based on Network Pharmacology, Molecular Docking and In Vitro Experiments
Source: Front Pharmacol. 2021 Jul 15;12:678685. doi: 10.3389/fphar.2021.678685 (PMC8320847; doi:10.3389/fphar.2021.678685)
Supplement: Supplementary file 1 [file DataSheet2.PDF]

Figure S1 Mass Spectrum of Quercetin

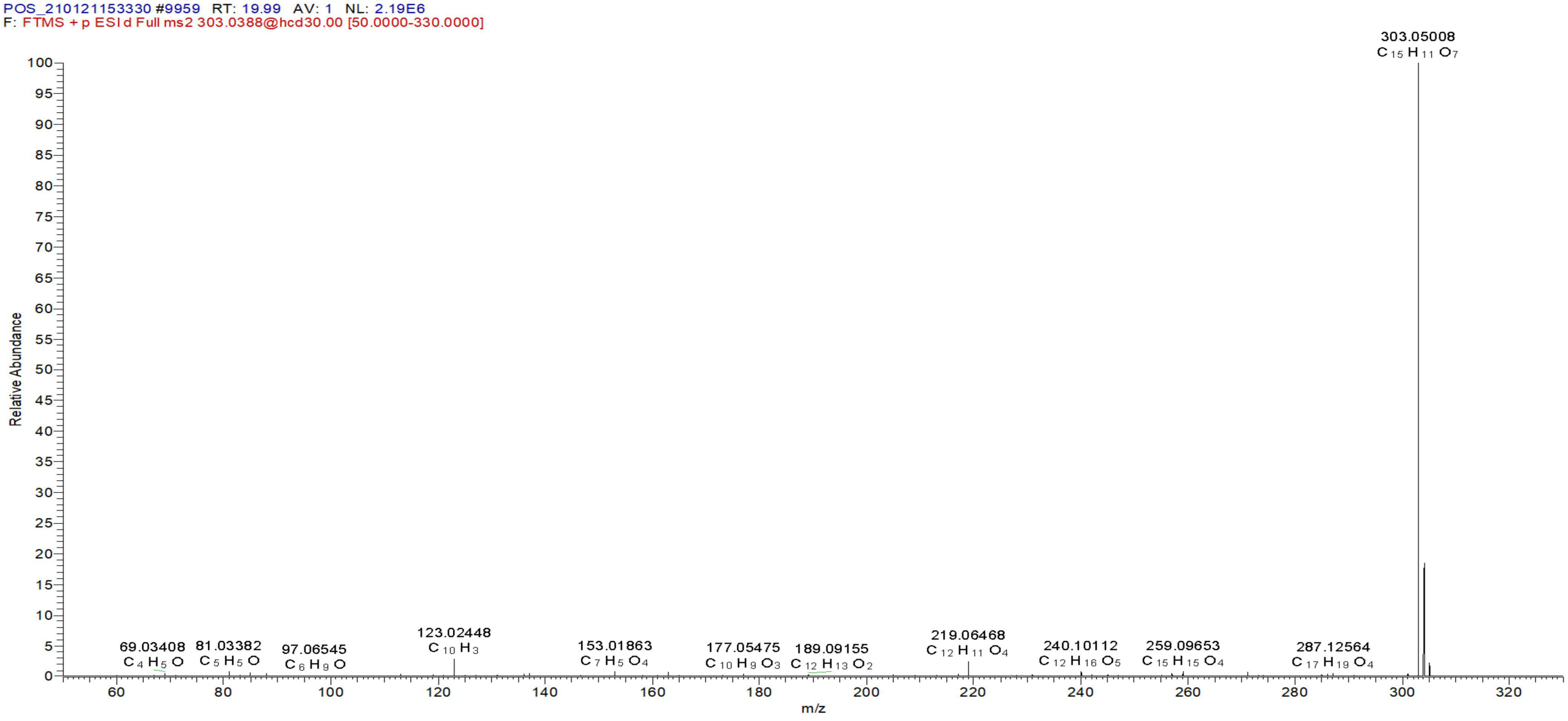

NO	TR min	ESI-MS	ESI-MS/MS	Molecular formula	Identity	Ref.
1	19.99	303.05008 $[M+H]^+$	153.01863	$C_{15}H_{10}O_7$	quercetin	Liu JL et al., 2016

Figure S2 Mass Spectrum of Beta-sitosterol

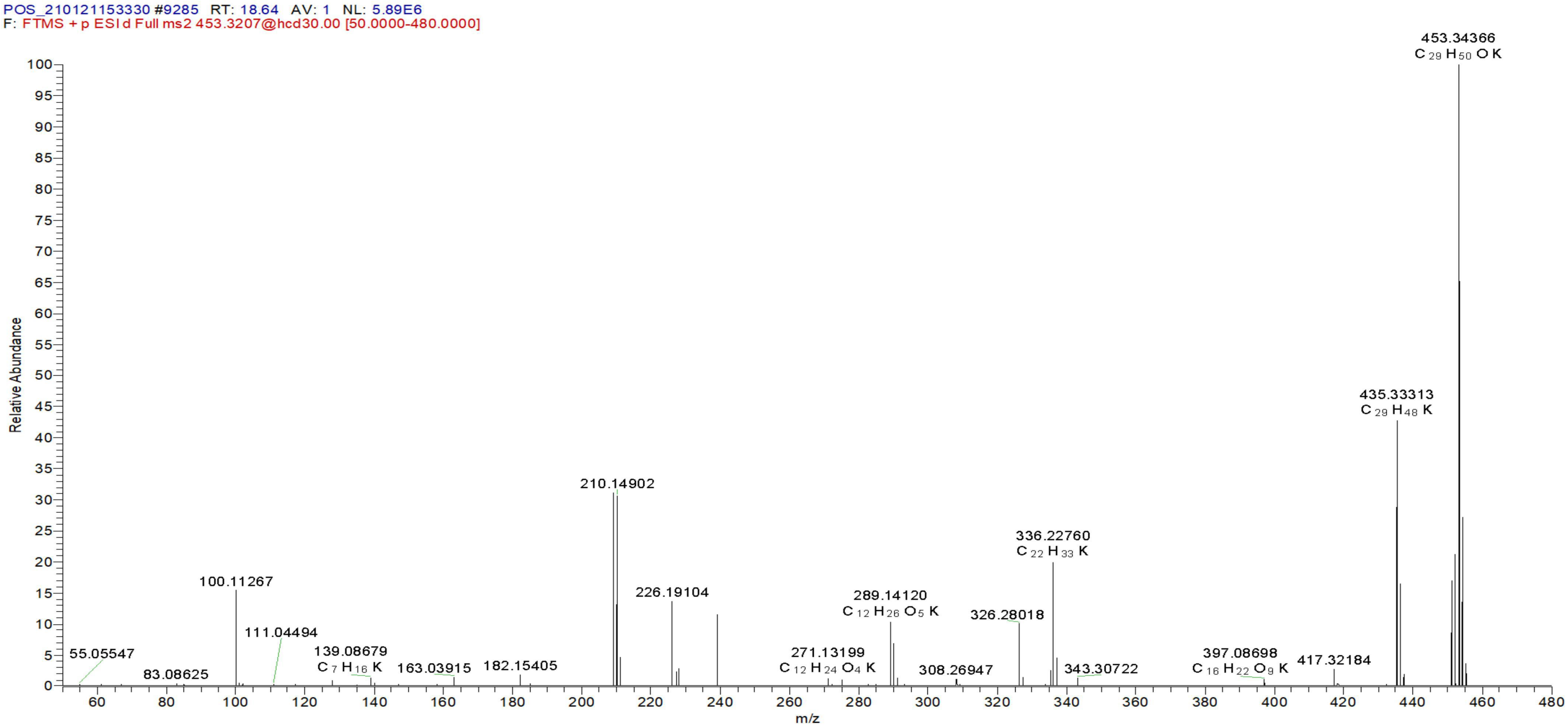

NO	TR min	ESI-MS	ESI-MS/MS	Molecular formula	Identity	Ref.
2	18.64	453.34366 $[M+K]^+$	139.08679	$C_{29}H_{50}O$	beta-sitosterol	Liu J., 2015

Figure S3 Mass Spectrum of Luteolin

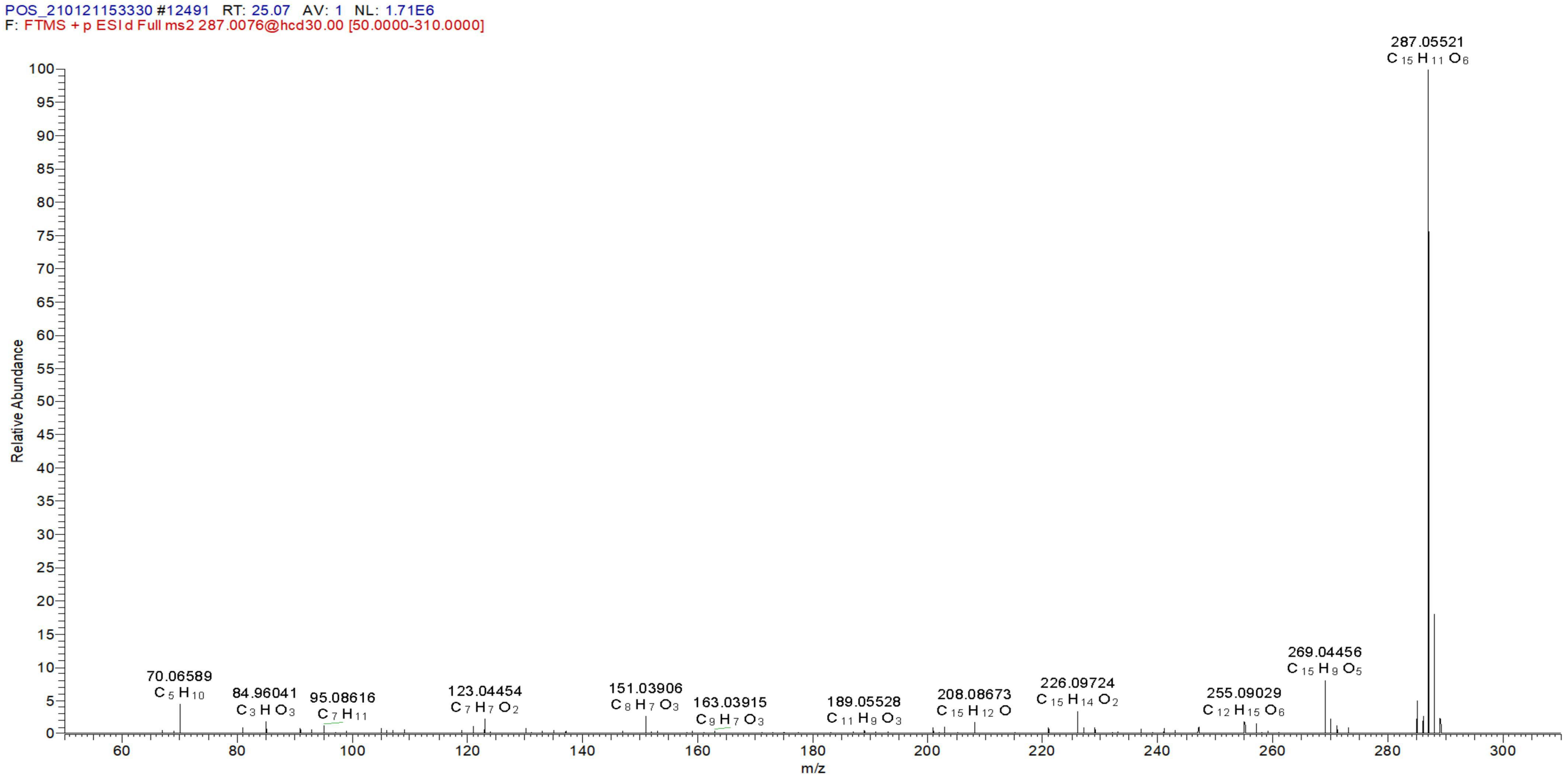

NO	TR min	ESI-MS	ESI-MS/MS	Molecular formula	Identity	Ref.
4	25.07	287.05521 $[M+H]^+$	255.09029, 151.03906	$C_{15}H_{10}O_6$	luteolin	Liu J., 2015

Figure S4 Mass Spectrum of Formononetin

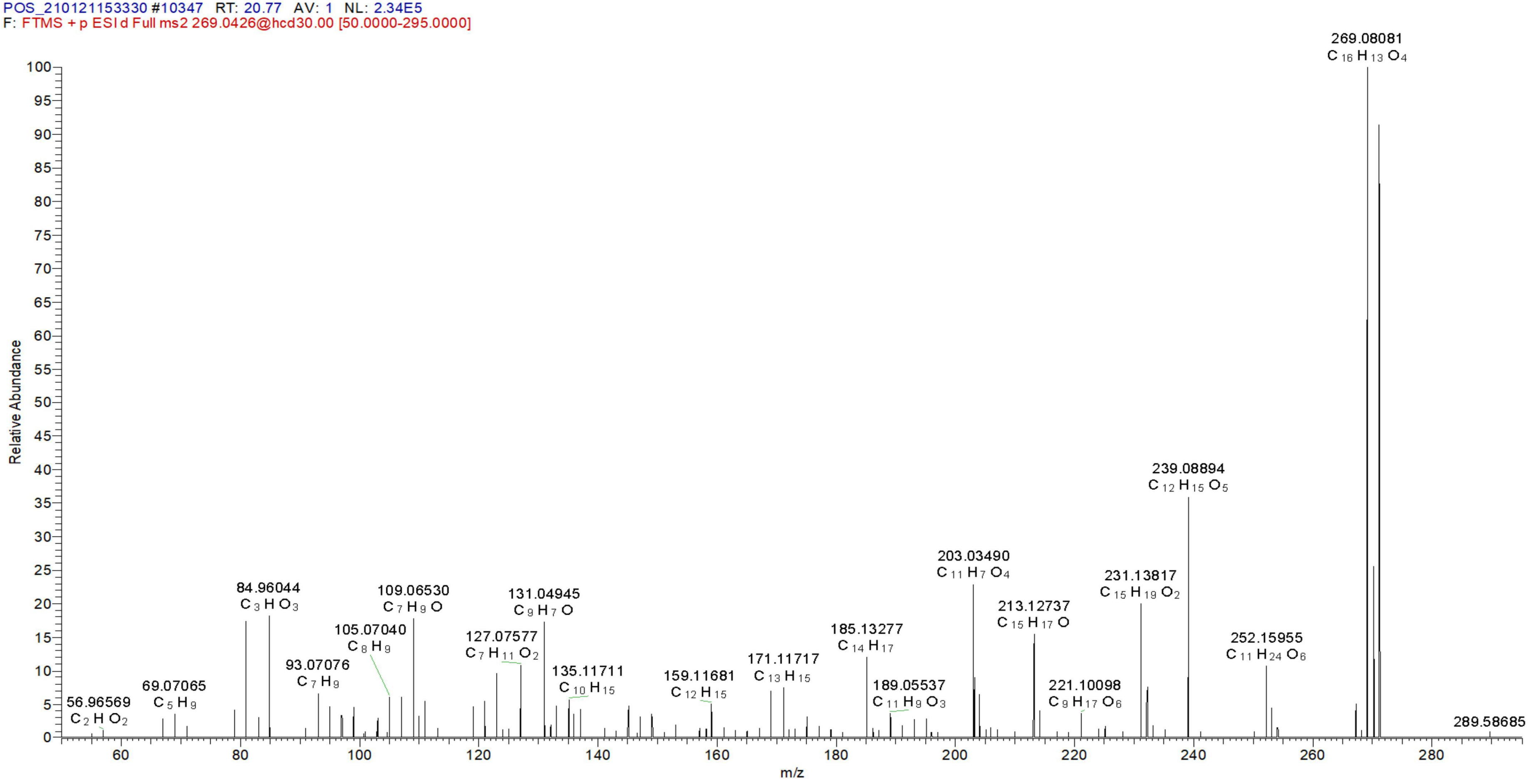

NO	TR min	ESI-MS	ESI-MS/MS	Molecular formula	Identity	Ref.
5	20.77	269.08081 $[M+H]^+$	213.12737	$C_{16}H_{12}O_4$	formononetin	Liu MH., 2015

Figure S5 Mass Spectrum of 8-Isopentenyl-kaempferol

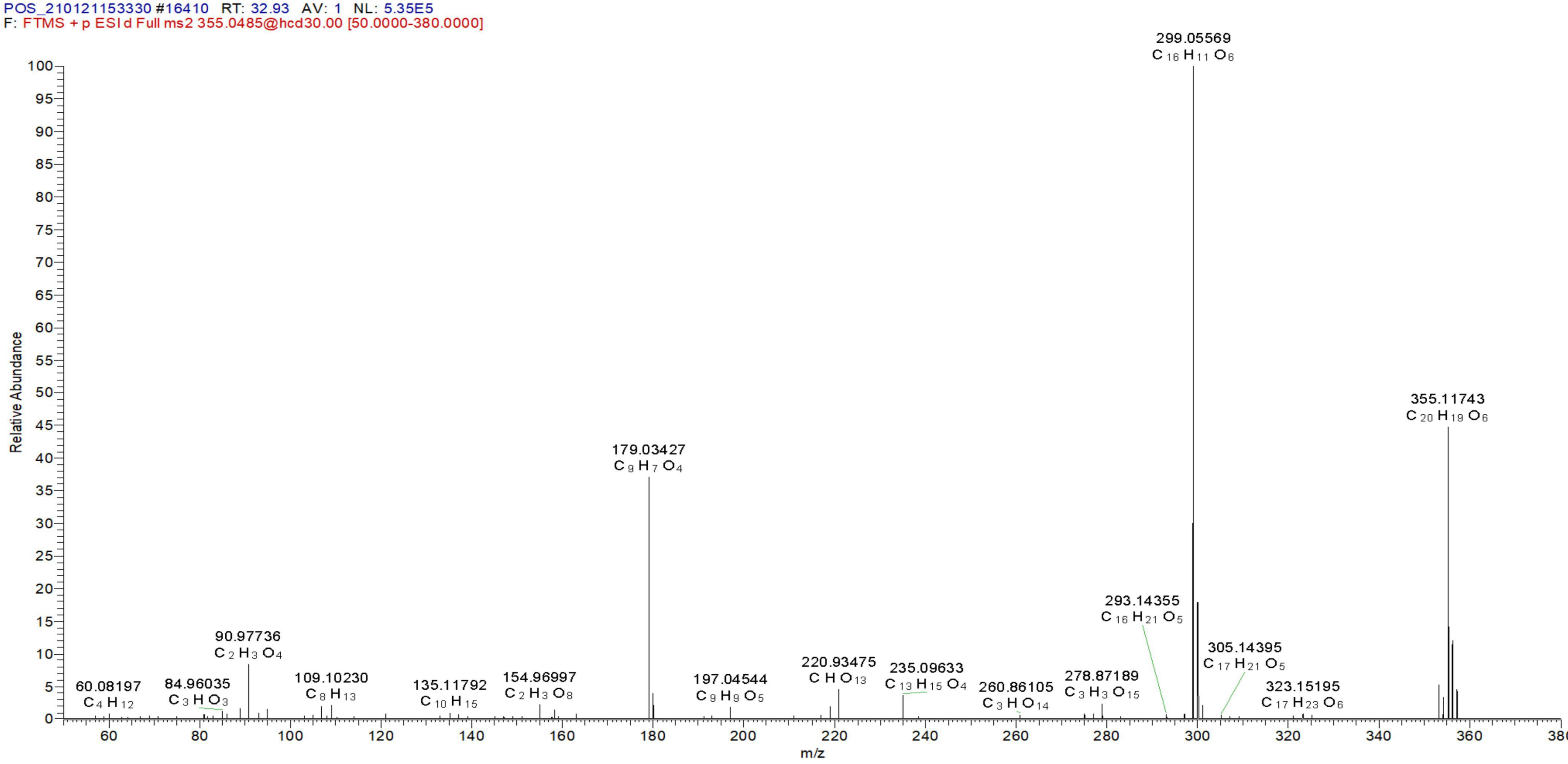

NO	TR min	ESI-MS	ESI-MS/MS	Molecular formula	Identity	Ref.
6	32.93	355.11743 $[M+H]^+$	179.03427	$C_{20}H_{18}O_6$	8-Isopentenyl-kaempferol	Liu J., 2015

Figure S6 Mass Spectrum of Aloe-emodin

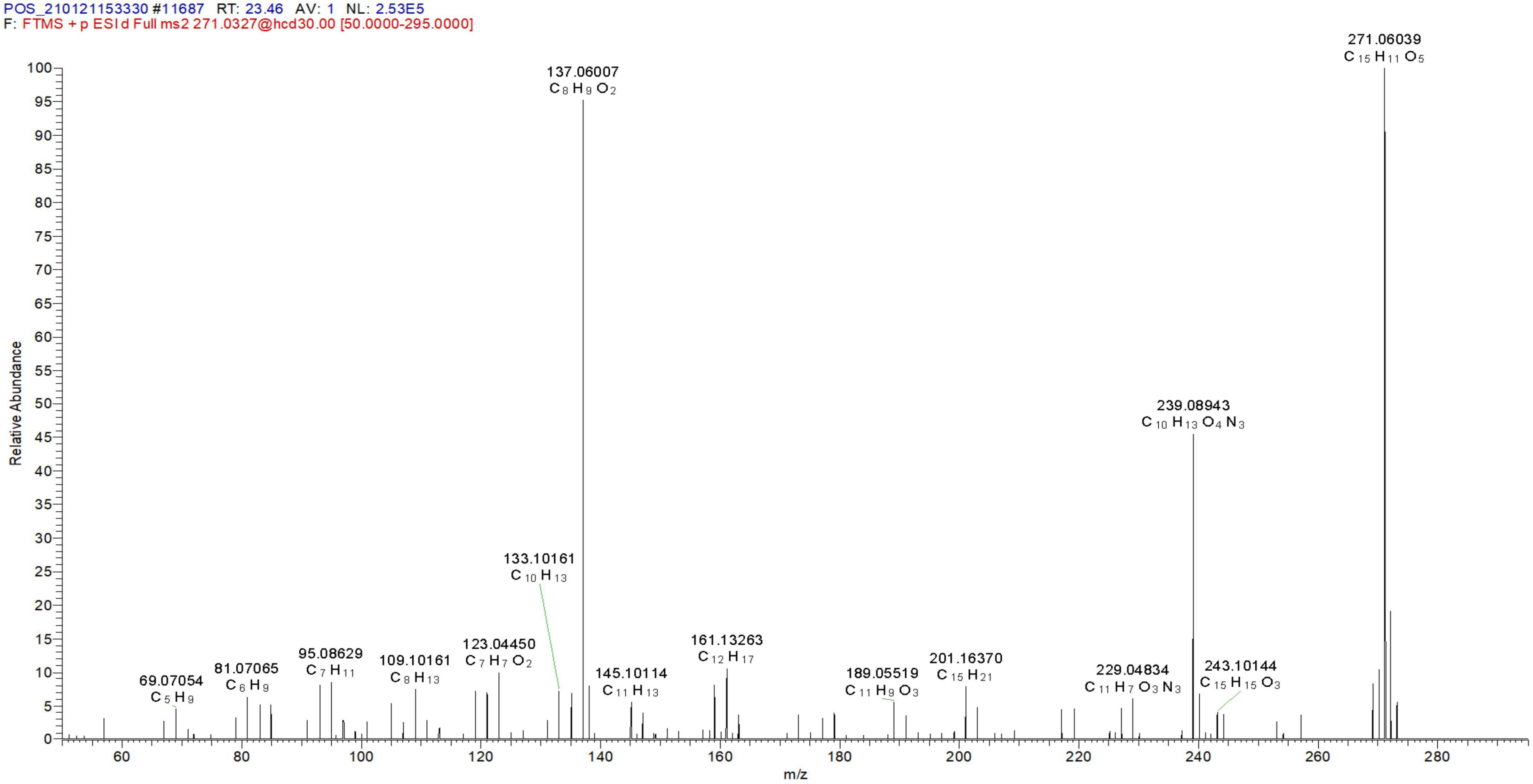

NO	TR min	ESI-MS	ESI-MS/MS	Molecular formula	Identity	Ref.
7	23.46	271.06039 $[M+H]^+$	137.06007	$C_{15}H_{10}O_5$	aloe-emodin	Liu J., 2015

Figure S7 Mass Spectrum of Phaseolin

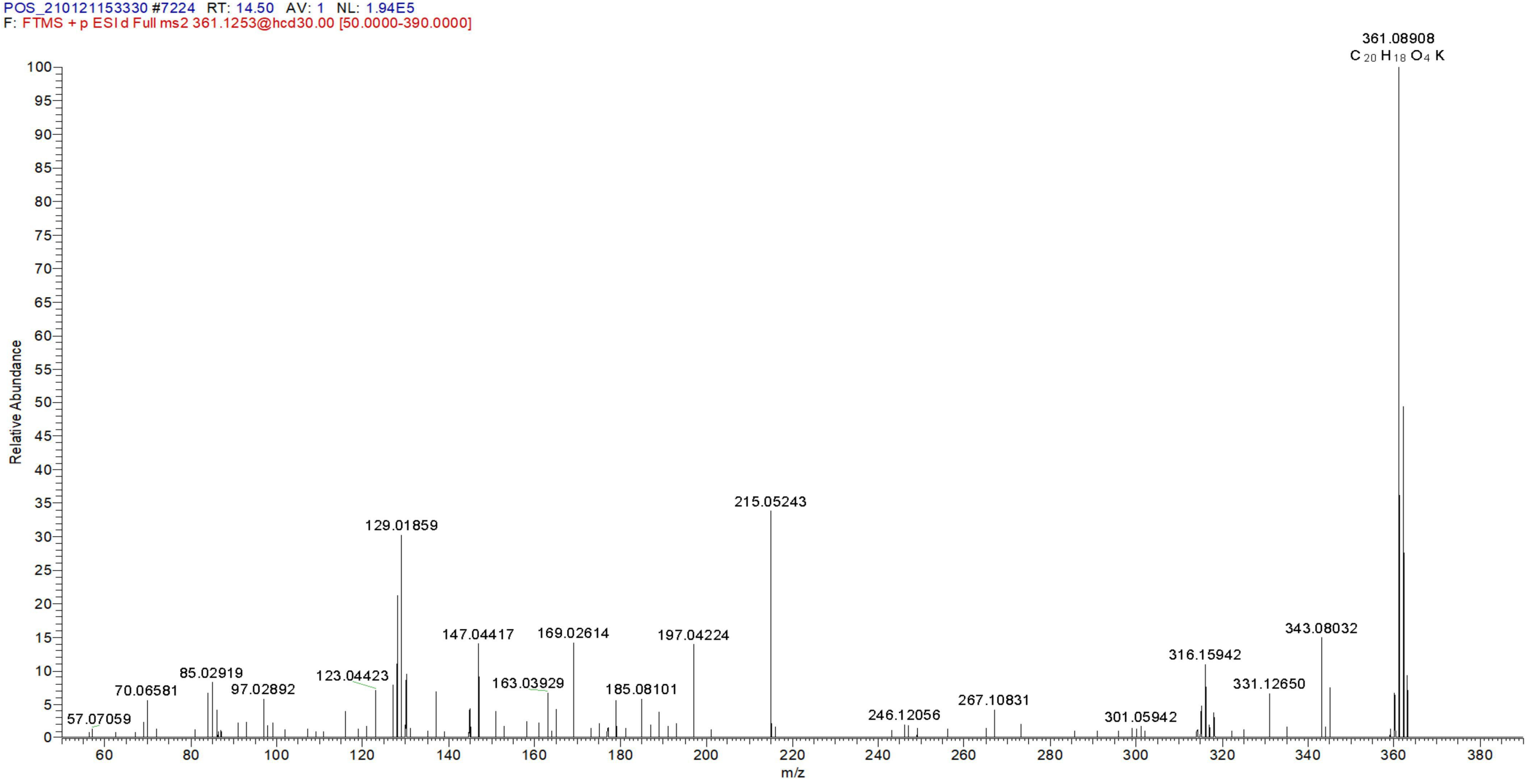

NO	TR min	ESI-MS	ESI-MS/MS	Molecular formula	Identity	Ref.
8	14.50	361.08908 $[M+K]^+$	215.05243, 129.01859	$C_{20}H_{18}O_4$	phaseolin	Liu J., 2015

Figure S8 Mass Spectrum of O-Isovalerylcolum bianetin

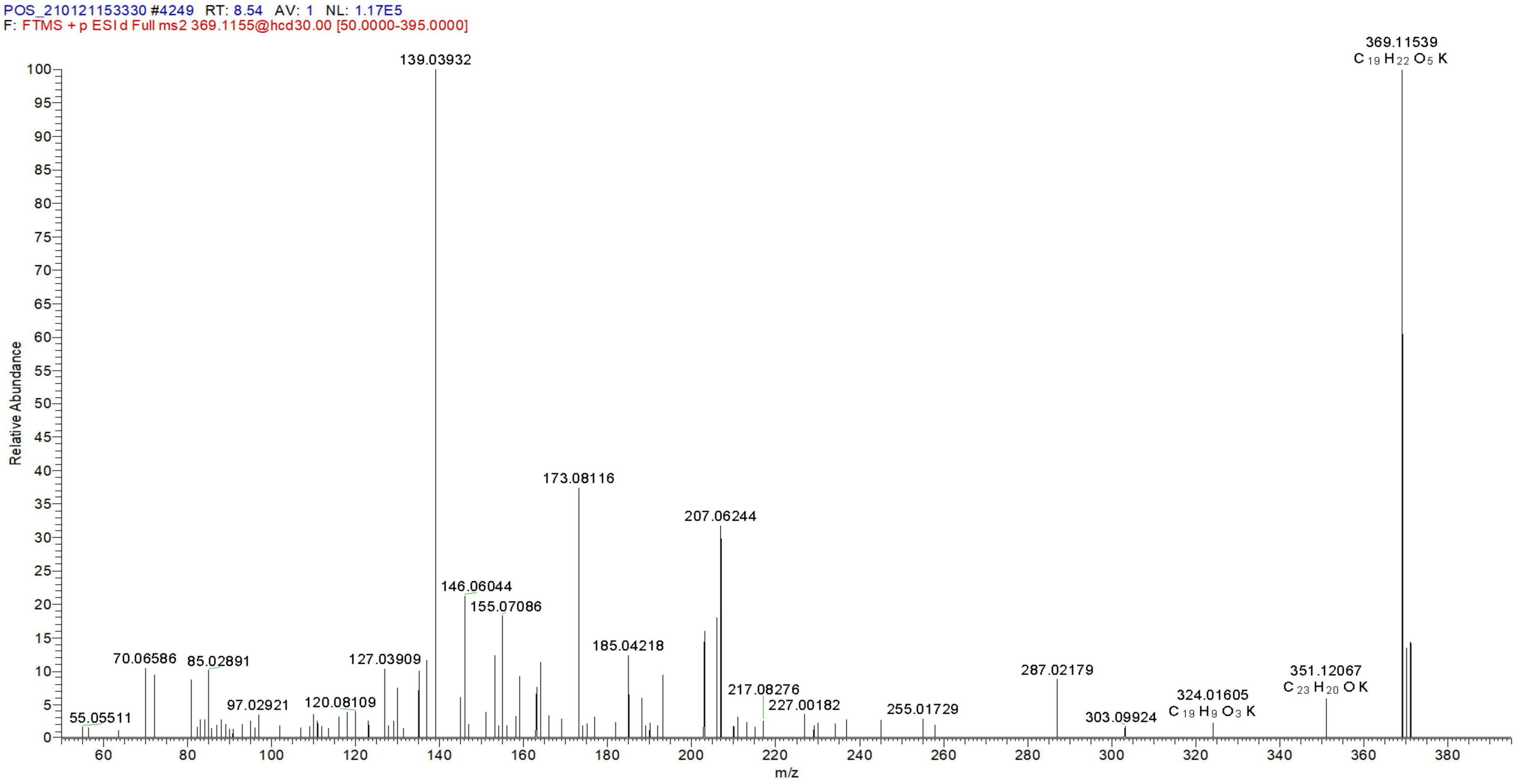

NO	TR min	ESI-MS	ESI-MS/MS	Molecular formula	Identity	Ref.
9	8.54	369.11539 $[M+K]^+$	351.12067	$C_{19}H_{22}O_5$	o-Isovalerylcolum bianetin	Liu J., 2015

Figure S9 Mass Spectrum of Wighteone

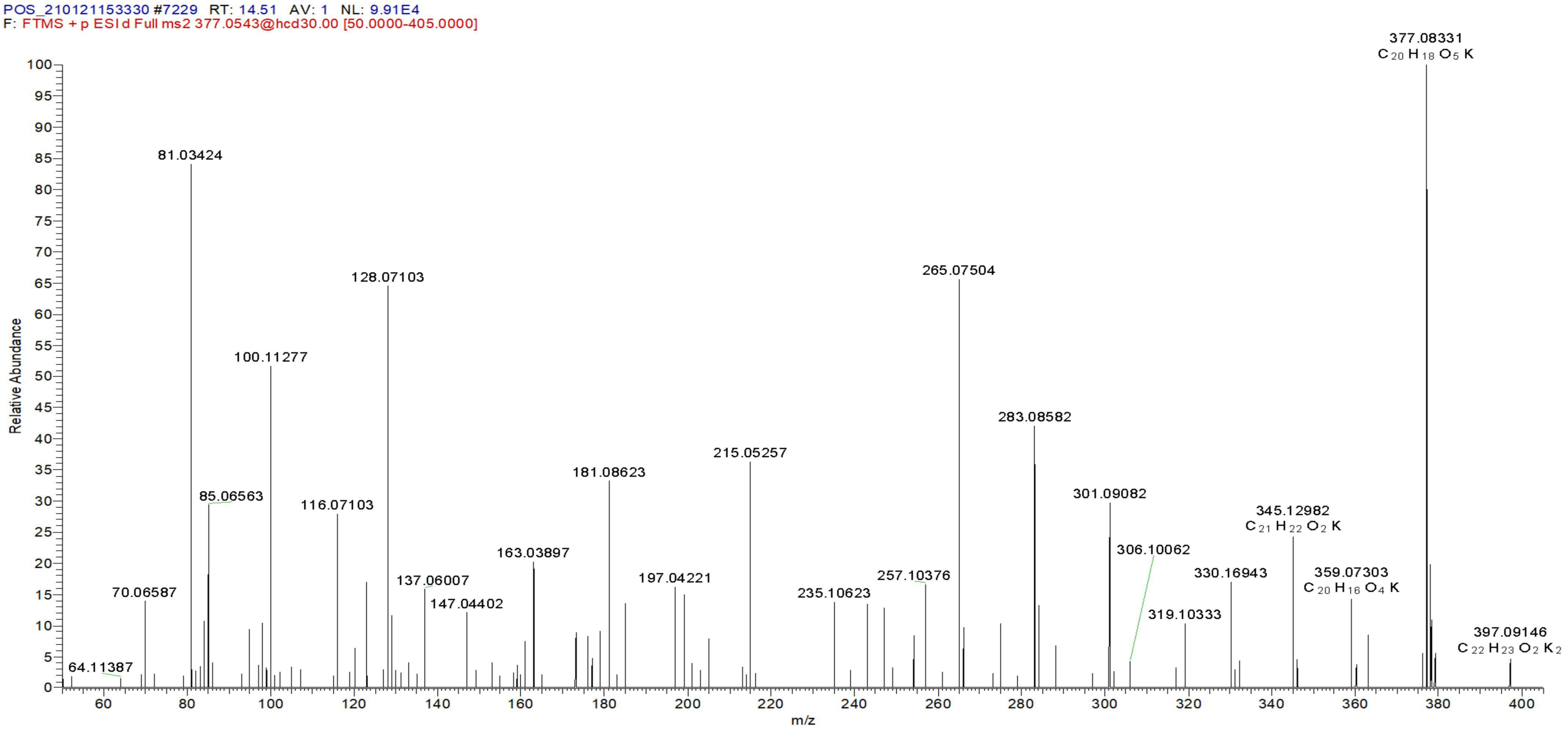

NO	TR min	ESI-MS	ESI-MS/MS	Molecular formula	Identity	Ref.
10	14.51	377.08331 $[M+K]^+$	359.07303, 345.12982	$C_{20}H_{18}O_5$	wighteone	Liu J., 2015

1 Figure S1 Mass Spectrum of Quercetin

2 Figure S2 Mass Spectrum of Beta-sitosterol

3 Figure S3 Mass Spectrum of Luteolin

4 Figure S4 Mass Spectrum of Formononetin

5 Figure S5 Mass Spectrum of 8-Isopentenyl-kaempferol

6 Figure S6 Mass Spectrum of Aloe-emodin

7 Figure S7 Mass Spectrum of Phaseolin

8 FigureS 8 Mass Spectrum of O-Isovalerylcolum bianetin

9 Figure S9 Mass Spectrum of Wighteone
