# Supplementary material for: Mechanism of Anti-Inflammatory and Antibacterial Effects of QingXiaoWuWei Decoction Based on Network Pharmacology, Molecular Docking and In Vitro Experiments
Source: Front Pharmacol. 2021 Jul 15;12:678685. doi: 10.3389/fphar.2021.678685 (PMC8320847; doi:10.3389/fphar.2021.678685)
Supplement: Supplementary file 2 [file DataSheet4.PDF]

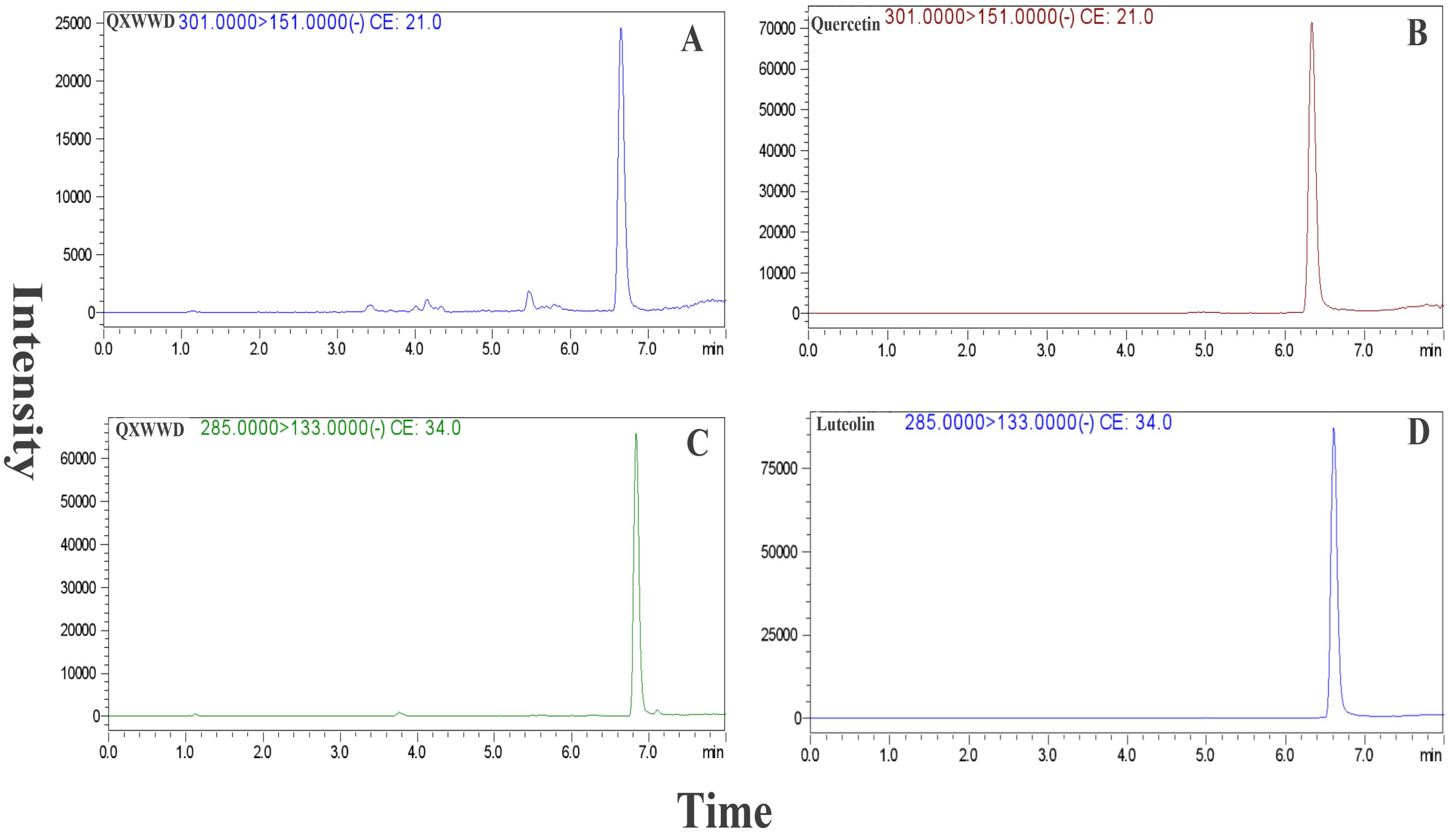

Figure S15 HPLC chromatograms of A: quercetin in QXWWD sample, B: quercetin standard , C: luteolin in QXWWD sample, B: luteolin standard
