# Supplementary material for: Mechanism of Anti-Inflammatory and Antibacterial Effects of QingXiaoWuWei Decoction Based on Network Pharmacology, Molecular Docking and In Vitro Experiments
Source: Front Pharmacol. 2021 Jul 15;12:678685. doi: 10.3389/fphar.2021.678685 (PMC8320847; doi:10.3389/fphar.2021.678685)
Supplement: Supplementary file 3 [file DataSheet3.PDF]

Figure S10 Quantitative Estimation of Antimicrobial Activity of QXWWD by MIC Method (μg/mL) ***Enterococcus faecalis*** **(ATCC29212)**

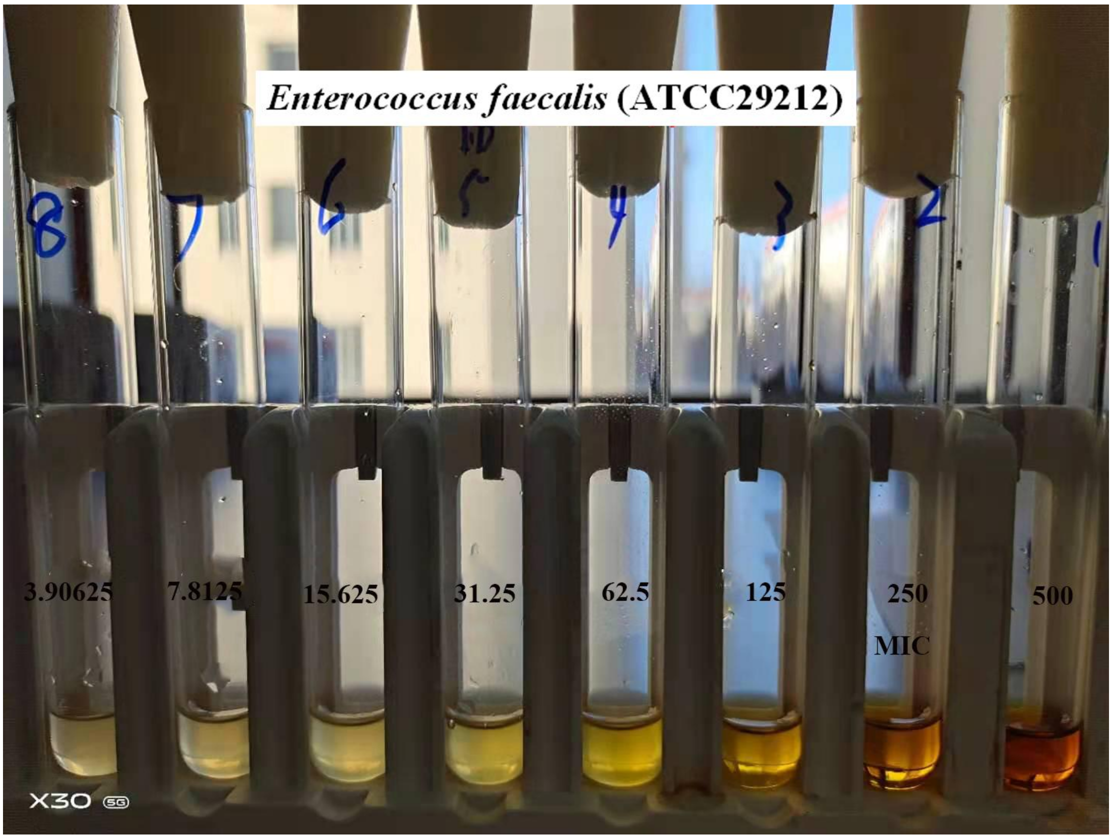

Figure S11 Quantitative Estimation of Antimicrobial Activity of QXWWD by MIC Method (μg/mL) *Staphylococcus aureus* (ATCC25923)

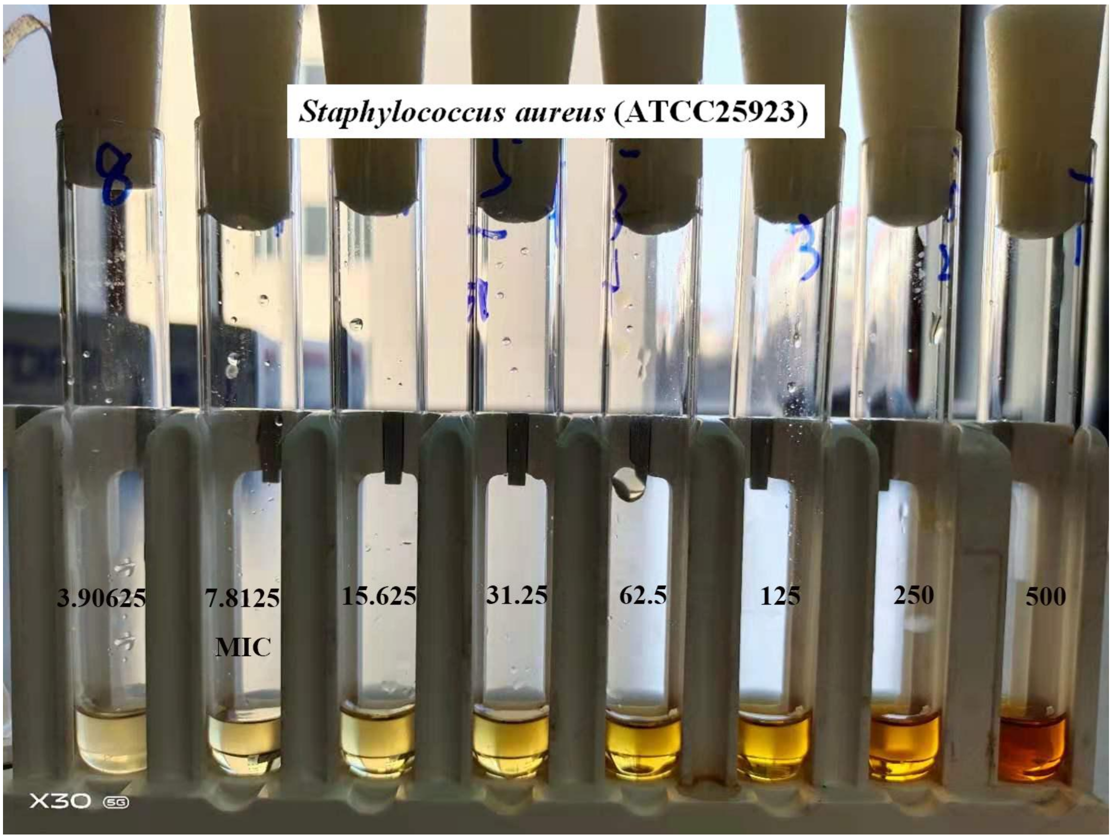

Figure S12 Quantitative Estimation of Antimicrobial Activity of QXWWD by MIC Method (μg/mL) *Staphylococcus aureus* (ATCC29213)

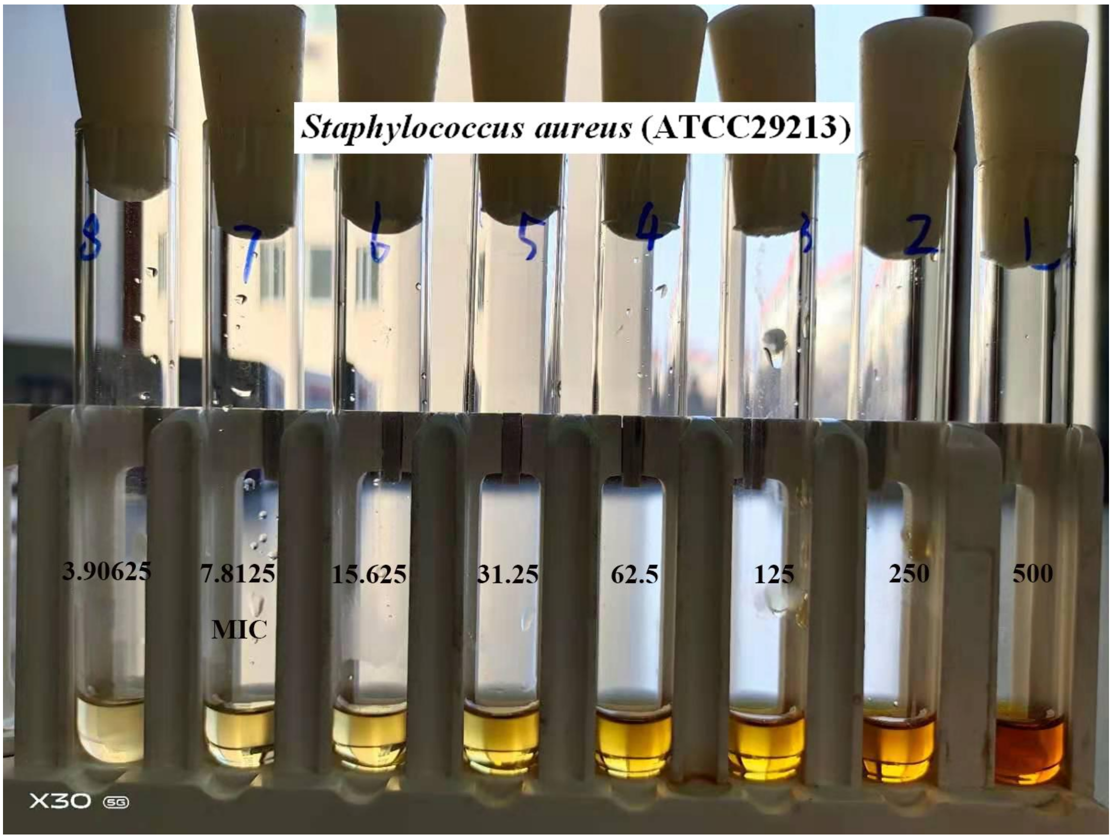

Figure S13 Quantitative Estimation of Antimicrobial Activity of QXWWD by MIC Method (μg/mL) *Staphylococcus aureus* (ATCC43300)

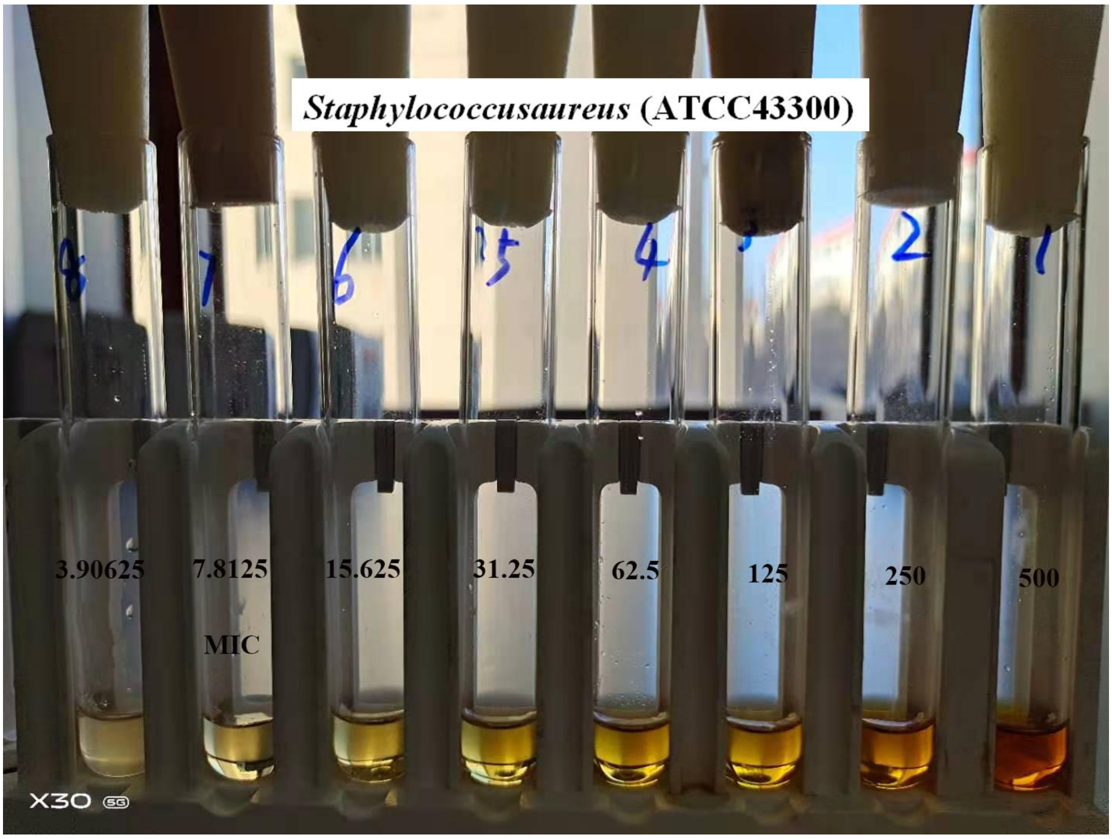

Figure S14 Quantitative Estimation of Antimicrobial Activity of QXWWD by MIC Method (μg/mL) *Streptococcus pneumoniae* (ATCC49619)

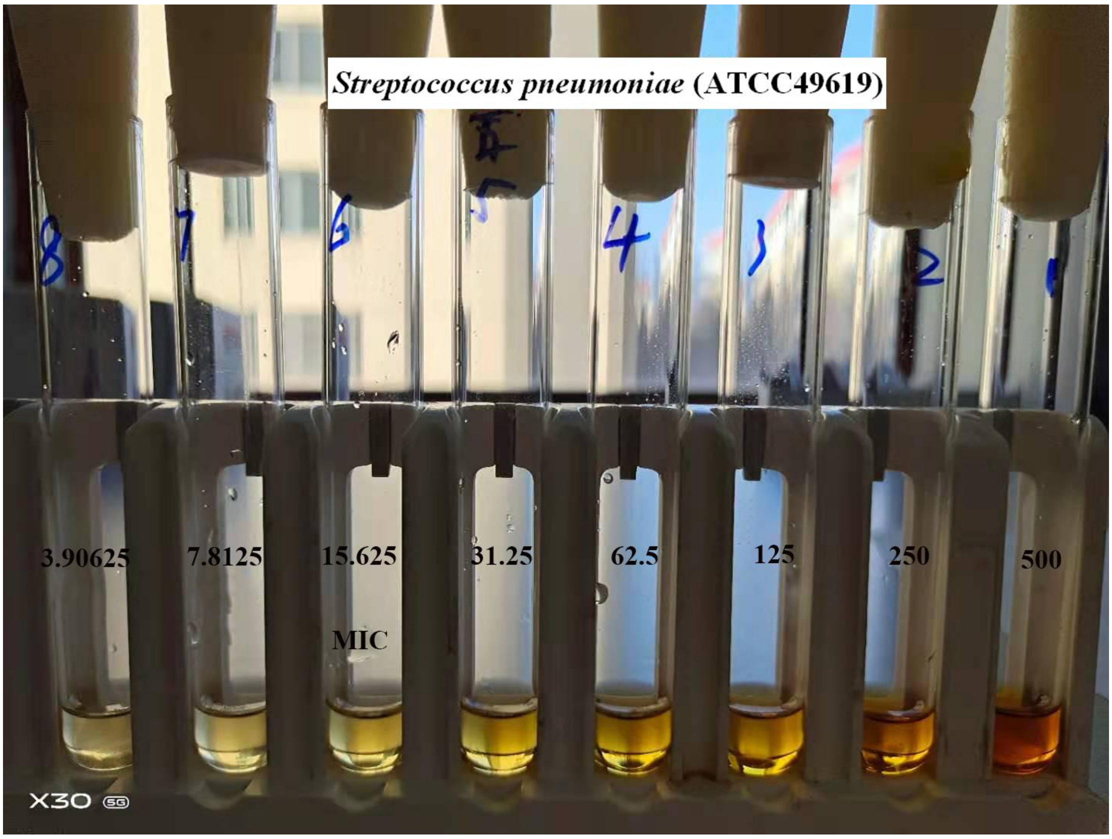

1 Figure S10 Quantitative Estimation of Antimicrobial Activity of QXWWD by MIC Method *Enterococcus faecalis* (ATCC29212)

2 Figure S11 Quantitative Estimation of Antimicrobial Activity of QXWWD by MIC Method *Staphylococcus aureus* (ATCC25923)

3 FigureS 12 Quantitative Estimation of Antimicrobial Activity of QXWWD by MIC Method *Staphylococcus aureus* (ATCC29213)

4 Figure S13 Quantitative Estimation of Antimicrobial Activity of QXWWD by MIC Method *Staphylococcus aureus* (ATCC43300)

5 Figure S14 Quantitative Estimation of Antimicrobial Activity of QXWWD by MIC Method *Streptococcus pneumoniae* (ATCC49619)
