# Supplementary material for: Mechanism of Anti-Inflammatory and Antibacterial Effects of QingXiaoWuWei Decoction Based on Network Pharmacology, Molecular Docking and In Vitro Experiments
Source: Front Pharmacol. 2021 Jul 15;12:678685. doi: 10.3389/fphar.2021.678685 (PMC8320847; doi:10.3389/fphar.2021.678685)
Supplement: Supplementary file 4 [file DataSheet1.PDF]

Table S1 Xylene-Induced Ear Swelling Test (Ear swelling rate=(M_R-M_L)/M_L×100%)

	Control group (0.5% CMC-Na)			Model group Xylene (0.02mL)			High dose group QXWWD (680 mg/kg)		
	M_R	M_L	Ear swelling rate	M_R	M_L	Ear swelling rate	M_R	M_L	Ear swelling rate
1	0.0068	0.0059	15.25%	0.0119	0.0067	77.61%	0.0067	0.0058	15.52%
2	0.0065	0.0067	0.00%	0.0100	0.0062	61.29%	0.0069	0.0062	11.29%
3	0.0071	0.0061	16.39%	0.0117	0.0055	112.73%	0.0057	0.0068	0.00%
4	0.0063	0.0075	0.00%	0.0131	0.0072	81.94%	0.0065	0.0073	0.00%
5	0.0064	0.0057	12.28%	0.0116	0.0064	81.25%	0.0076	0.0069	10.14%
6	0.0069	0.0063	9.52%	0.0098	0.0060	63.33%	0.0068	0.0064	6.25%
7	0.0066	0.0058	13.79%	0.0110	0.0074	48.65%	0.0064	0.0070	0.00%
8	0.0063	0.0070	0.00%	0.0104	0.0059	76.27%	0.0065	0.0072	0.00%
9	0.0067	0.0053	26.42%	0.0114	0.0063	80.95%	0.0081	0.0067	20.90%
10	0.0064	0.0066	0.00%	0.0114	0.0068	67.65%	0.0069	0.0061	13.11%
**Mean**			**9.37%**			**75.17%****[**]**			**7.72%**[##]
**SD**			**9.15%**			**17.08%**			**7.62%**
	Medium dose group QXWWD (340 mg/kg)			Low dose group QXWWD (170 mg/kg)			Aspirin (200 mg/kg)		
1	M_R	M_L	Ear swelling rate	M_R	M_L	Ear swelling rate	M_R	M_L	Ear swelling rate
2	0.0082	0.0063	30.16%	0.0099	0.0066	50.00%	0.0066	0.0068	0.00%
3	0.0091	0.0069	31.88%	0.0094	0.0057	64.91%	0.0070	0.0072	0.00%
4	0.0091	0.0072	26.39%	0.0076	0.0063	20.63%	0.0071	0.0060	18.33%
5	0.0088	0.0056	57.14%	0.0101	0.0080	26.25%	0.0068	0.0056	21.43%
6	0.0084	0.0059	42.37%	0.0107	0.0064	67.19%	0.0066	0.0063	4.76%
7	0.0095	0.0067	41.79%	0.0099	0.0060	65.00%	0.0071	0.0075	0.00%
8	0.0088	0.0058	51.72%	0.0109	0.0072	51.39%	0.0067	0.0079	0.00%
9	0.0090	0.0072	25.00%	0.0098	0.0065	50.77%	0.0072	0.0065	10.77%
10	0.0080	0.0060	33.33%	0.0095	0.0074	28.38%	0.0072	0.0061	18.03%
1	0.0087	0.0065	33.85%	0.0084	0.0058	44.83%	0.0086	0.0078	10.26%
**Mean**			**37.36%**[##]			**46.93%**[##]			**8.36%**[##]
**SD**			**10.68%**			**16.90%**			**8.60%**

**P <0.01 in contrast with the control group, ##P <0. 01 in contrast with the model group

Table S2 White Blood Cell (WBC) Counts Measurement (×10^9 cells /L)

	Control group (0.5% CMC-Na)	Model group Xylene (0.02mL)	High dose group QXWWD (680 mg/kg)	Medium dose group QXWWD (340 mg/kg)	Low dose group QXWWD (170 mg/kg)	Aspirin 200 mg/kg
1	4.58	7.99	4.66	5.63	6.88	4.56
2	4.77	8.39	4.75	4.99	6.32	4.65
3	4.32	8.13	4.39	5.34	6.12	4.29
4	4.65	8.77	4.56	5.78	5.78	4.53
5	4.21	7.19	4.21	6.11	5.89	4.51
6	4.19	7.89	4.29	5.08	5.45	4.59
7	4.33	8.12	4.31	5.69	6.03	4.32
8	4.18	8.33	4.22	6.03	6.76	4.32
9	4.53	7.68	4.33	5.19	6.79	4.13
10	4.22	7.59	4.21	5.88	5.93	4.22
**Mean**	**4.40**	**8.01** **	**4.39** $^{\#\#}$	**5.57** $^{\#\#}$	**6.20** $^{\#\#}$	**4.41** $^{\#\#}$
**SD**	**0.22**	**0.45**	**0.20**	**0.40**	**0.48**	**0.18**

$^{**}P<0.01$ in contrast with the control group, $^{\#\#}P<0.\ 01$ in contrast with the model group

Table S3 Antibacterial Activity Test (Inhibition zone diameter cm)

		Staphylococcus aureus ATCC29213	*Staphylococcus aureus* ATCC25923	*Staphylococcus aureus* ATCC43300	*Enterococcus faecalis* ATCC29212	*Streptococcus pneumoniae* ATCC49619
Positive control (VA 30 μg)	1	17.3	16.8	16.2	14.6	26.8
	2	18.1	16.1	15.7	15.2	25.7
	3	18.4	15.6	15.9	15.3	25.6
	**Mean**	**17.9**	**16.2**	**15.9**	**15.0**	**26.0**
	**SD**	**0.6**	**0.6**	**0.3**	**0.4**	**0.7**
QXWWD (1g/mL)	1	8.5	8.8	9.2	6.8	10.5
	2	8.6	9.2	9.3	6.6	10.1
	3	8.9	8.6	8.6	7.2	9.8
	4	9.3	8.9	8.7	7.5	9.4
	5	9.4	9.4	8.9	6.9	10.4
	6	9.6	9.2	9.0	7.0	9.6
	7	9.3	9.6	9.4	7.3	9.9
	8	8.6	8.7	8.5	6.6	9.7
	9	9.3	9.4	9.2	7.4	10.7
	10	9.5	8.7	9.3	7.1	10.1
	11	9.9	9.0	8.7	6.7	10.0
	12	8.9	9.1	9.0	7.3	9.6
	**Mean**	**9.2** $^{**/\#\#}$	**9.1** $^{**/\#\#}$	**9.0** $^{**/\#\#}$	**7.0** $^{**/\#\#}$	**10.0** $^{**/\#\#}$
	**SD**	**0.4**	**0.3**	**0.3**	**0.3**	**0.4**
Negtive control (normal saline)	1	6.0	6.0	6.0	6.0	6.0
	2	6.0	6.0	6.0	6.0	6.0
	3	6.0	6.0	6.0	6.0	6.0
	**Mean**	**6.0**	**6.0**	**6.0**	**6.0**	**6.0**
	**SD**	**0.0**	**0.0**	**0.0**	**0.0**	**0.0**

$^{**}P$ <0.01 in contrast with the positive control group, $^{\#\#}P$ <0. 01 in contrast with the negtive control group

Table S4 Antibacterial Activity Test (Inhibition zone diameter cm)

		Escherichia coli ATCC25922	*Pseudomonas aeruginosa* ATCC27853	*Enterobacter cloacae* ATCC700323	*Klebsiella pneumoniae* ATCC700603	*Streptococcus pyogenes* ATCC19615
Positive control (IPM 10 μg)	1	25.7	20.3	25.6	25.1	18.2
	2	26.6	19.9	24.9	24.6	17.6
	3	26.1	19.7	24.6	25.3	17.9
	**Mean**	**26.1**	**20.0**	**25.0**	**25.0**	**17.9**
	**SD**	**0.5**	**0.3**	**0.5**	**0.4**	**0.3**
QXWWD (1g/mL)	1	6.0	6.0	6.0	6.0	6.0
	2	6.0	6.0	6.0	6.0	6.0
	3	6.0	6.0	6.0	6.0	6.0
	4	6.0	6.0	6.0	6.0	6.0
	5	6.0	6.0	6.0	6.0	6.0
	6	6.0	6.0	6.0	6.0	6.0
	7	6.0	6.0	6.0	6.0	6.0
	8	6.0	6.0	6.0	6.0	6.0
	9	6.0	6.0	6.0	6.0	6.0
	10	6.0	6.0	6.0	6.0	6.0
	11	6.0	6.0	6.0	6.0	6.0
	12	6.0	6.0	6.0	6.0	6.0
	**Mean**	**6.0** **	**6.0** **	**6.0** **	**6.0** **	**6.0** **
	**SD**	**0.0**	**0.0**	**0.0**	**0.0**	**0.0**
Negtive control (normal saline)	1	6.0	6.0	6.0	6.0	6.0
	2	6.0	6.0	6.0	6.0	6.0
	3	6.0	6.0	6.0	6.0	6.0
	**Mean**	**6.0**	**6.0**	**6.0**	**6.0**	**6.0**
	**SD**	**0.0**	**0.0**	**0.0**	**0.0**	**0.0**

**P <0.01 in contrast with the positive control group, ##P <0. 01 in contrast with the negtive control group

Table S5 Cell Culture and Viability Assay

	Absorbance at 490 nm									
Concentrations of DXWWD(μg/mL)	500	250	125	62.5	31.25	15.625	7.8125	3.90625	1.953125	0
1	0.280	0.451	0.809	1.131	1.252	1.284	1.701	1.225	1.191	1.254
2	0.295	0.445	0.743	1.257	1.497	1.380	1.358	1.294	1.169	1.429
3	0.284	0.464	0.862	1.258	1.336	1.427	1.270	1.386	1.377	1.263
4	0.284	0.465	0.710	1.332	1.472	1.429	1.421	1.336	1.394	1.071
5	0.304	0.433	0.881	1.255	1.341	1.343	1.369	1.361	1.553	0.922
6	0.286	0.446	0.830	1.361	1.283	1.314	1.250	1.276	1.198	0.997
**Mean**	**0.289**	**0.451**	**0.806**	**1.266**	**1.364**	**1.363**	**1.395**	**1.313**	**1.314**	**1.156**
**SD**	**0.009**	**0.012**	**0.067**	**0.080**	**0.100**	**0.060**	**0.163**	**0.059**	**0.153**	**0.191**
**Survival rate**	**25.0%**	**39.0%**	**69.7%**	**109.5%**	**117.9%**	**117.9%**	**120.7%**	**113.6%**	**113.6%**	**100.0%**

Table S6 Pro-inflammatory Cytokines Measurement (ng/L)

	Control group (0.1% DMSO)	Model group LPS (1 μg/mL)	High dose group QXWWD (80 μg/mL)	Medium dose group QXWWD (40 μg/mL)	Low dose group QXWWD (20 μg/mL)
IL-1					
1	145.32	265.44	158.22	198.54	233.56
2	133.58	280.31	151.93	183.64	224.68
3	126.31	253.69	144.26	179.35	215.82
4	120.56	251.88	161.87	168.24	210.22
5	135.47	258.54	156.18	196.77	209.38
6	121.89	262.81	150.48	208.43	227.51
**Mean**	**130.52**	**262.11** **	**153.82** ##	**189.16** ##	**220.20** ##
**SD**	**9.43**	**10.31**	**6.26**	**14.71**	**9.88**
IL-6					
1	105.43	197.28	124.51	142.56	164.53
2	101.94	183.47	128.66	149.26	148.69
3	98.25	166.52	108.49	133.57	175.23
4	94.61	179.88	121.63	148.77	166.28
5	82.83	186.34	129.43	134.92	179.31
6	103.56	196.58	115.47	148.25	159.58
**Mean**	**97.77**	**185.01** **	**121.37** ##	**142.89** ##	**165.60** #
**SD**	**8.28**	**11.46**	**8.11**	**7.13**	**11.00**
TNF-α					
1	304.26	588.49	411.87	478.29	549.26
2	288.38	637.58	423.26	493.77	558.38
3	286.54	615.29	402.57	525.24	537.64
4	272.65	588.92	386.55	501.48	519.47
5	297.29	624.31	399.23	491.26	558.91
6	317.46	630.55	427.84	475.32	539.46
**Mean**	**294.43**	**614.19** **	**408.55** ##	**494.23** ##	**543.85** ##
**SD**	**15.55**	**21.06**	**15.53**	**18.08**	**14.96**

***P* <0.01 in contrast with the control group, ##*P* <0. 01, #*P* <0. 05 in contrast with the model group

Table S7 Skin Irritation Scores of Normal Skin Control Group

Animal number	1				2				3			
	1h	24h	48h	72h	1h	24h	48h	72h	1h	24h	48h	72h
Erythema	0	0	0	0	0	0	0	0	0	0	0	0
Oedema	0	0	0	0	0	0	0	0	0	0	0	0
Animal number	4				5				6			
	1h	24h	48h	72h	1h	24h	48h	72h	1h	24h	48h	72h
Erythema	0	0	0	0	0	0	0	0	0	0	0	0
Oedema	0	0	0	0	0	0	0	0	0	0	0	0

Table S8 Skin Irritation Scores of Normal Skin Drug Group

Animal number	1				2				3			
	1h	24h	48h	72h	1h	24h	48h	72h	1h	24h	48h	72h
Erythema	0	0	0	0	0	0	0	0	0	0	0	0
Oedema	0	0	0	0	0	0	0	0	0	0	0	0
Animal number	4				5				6			
	1h	24h	48h	72h	1h	24h	48h	72h	1h	24h	48h	72h
Erythema	0	0	0	0	0	0	0	0	0	0	0	0
Oedema	0	0	0	0	0	0	0	0	0	0	0	0

Table S9 Skin Irritation Scores of Damage Skin Control Group

Animal number	7				8				9			
	1h	24h	48h	72h	1h	24h	48h	72h	1h	24h	48h	72h
Erythema	**1**	**1**	0	0	0	0	0	0	**1**	**1**	0	0
Oedema	0	0	0	0	0	0	0	0	**1**	0	0	0
Animal number	10				11				12			
	1h	24h	48h	72h	1h	24h	48h	72h	1h	24h	48h	72h
Erythema	0	0	0	0	**1**	0	0	0	0	0	0	0
Oedema	**1**	0	0	0	0	0	0	0	**1**	0	0	0

Table S10 Skin Irritation Scores of Damage Skin Drug Group

Animal number	7				8				9			
	1h	24h	48h	72h	1h	24h	48h	72h	1h	24h	48h	72h
Erythema	0	0	0	0	0	0	0	0	0	0	0	0
Oedema	0	0	0	0	0	0	0	0	0	0	0	0
Animal number	10				11				12			
	1h	24h	48h	72h	1h	24h	48h	72h	1h	24h	48h	72h
Erythema	0	0	0	0	0	0	0	0	0	0	0	0
Oedema	0	0	0	0	0	0	0	0	0	0	0	0

Table S11 Skin Irritation Scores

Group	Normal Skin Control Group (0.5% CMC-Na)				Normal Skin Drug Group QXWWD (680 mg/kg)				Damage Skin Control Group (0.5% CMC-Na)				Damage Skin Drug Group QXWWD (680 mg/kg)			
Time	1h	24h	48h	72h	1h	24h	48h	72h	1h	24h	48h	72h	1h	24h	48h	72h
Total scores	0	0	0	0	0	0	0	0	6	2	0	0	0	0	0	0
Average scores	0	0	0	0	0	0	0	0	**1**	**0.3**	0	0	0	0	0	0

Table S12 Docking Affinity

Target	Mol Name	Docking Affinity
RELA	quercetin	-7.7
	matrine	-6.6
	luteolin	-7.7
NFKBIA	quercetin	-7.7
	luteolin	-7.8
MYC	quercetin	-7.3
	aloe-emodin	-7.4
	matrine	-7.6
MAPK1	quercetin	-8.2
	luteolin	-7.9
MAPK14	8-Isopentenyl-kaempferol	-8.8
	Wighteone	-8.4
	formononetin	-8.5
	Phaseolin	-8.6
ESR1	(2R)-5,7-dihydroxy-2-(4-hydroxyphenyl)chroman-4-one	-8.6
	Toralactone	-7.6
	(-)-catechin	-7.9
	8-Isopentenyl-kaempferol	-7.6
	Wighteone	-7.1
	formononetin	-8.2
	Glyceollin	-8.1
	kushenin	-8.6
	leachianone,g	-7.9
	Phaseolin	-7.7
	Prangenidin	-8.3
	cnidimol B	-8.3
	O-Acetylcolumbianetin	-8.9
NR3C1	(2R)-5,7-dihydroxy-2-(4-hydroxyphenyl)chroman-4-one	-8.6
AKT1	quercetin	-9.8
	luteolin	-9.8
TP53	quercetin	-8.3
	aloe-emodin	-8.4
	luteolin	-8.4
JUN	beta-sitosterol	-7.6
	quercetin	-8.8
	rhein	-9.6
	formononetin	-8.3
	luteolin	-9
FOS	quercetin	-10.1

1 Table S1 Xylene-Induced Ear Swelling Test

2 Table S2 White Blood Cell (WBC) Counts Measurement

3 Table S3 Antibacterial Activity Test

4 Table S4 Antibacterial Activity Test

5 Table S5 Cell Culture and Viability Assay

6 Table S6 Pro-inflammatory Cytokines Measurement

7 Table S7 Skin Irritation Scores of Normal Skin Control Group

8 Table S8 Skin Irritation Scores of Normal Skin Drug Group

9 Table S9 Skin Irritation Scores of Damage Skin Control Group

10 Table S10 Skin Irritation Scores of Damage Skin Drug Group

11 Table S11 Skin Irritation Scores

12 Table S12 Docking Affinity
